# OVOL1 inhibits breast cancer cell invasion by enhancing the degradation of TGF-β type I receptor

**DOI:** 10.1038/s41392-022-00944-w

**Published:** 2022-04-29

**Authors:** Chuannan Fan, Qian Wang, Gerard van der Zon, Jiang Ren, Cedrick Agaser, Roderick C. Slieker, Prasanna Vasudevan Iyengar, Hailiang Mei, Peter ten Dijke

**Affiliations:** 1 Department of Cell and Chemical Biology, Leiden University Medical Center, Leiden, 2300 RC The Netherlands; 2 Oncode Institute, Leiden University Medical Center, Leiden, 2300 RC The Netherlands; 3 Department of Biomedical Data Sciences, Sequencing Analysis Support Core, Leiden University Medical Center, Leiden, 2300 RC The Netherlands; 4 Department of Epidemiology and Data Science, Amsterdam Public Health Institute, Amsterdam Cardiovascular Sciences Institute, Amsterdam UMC, location VUmc, Amsterdam, 1081 HV The Netherlands

**Keywords:** Breast cancer, Metastasis

## Abstract

Ovo-like transcriptional repressor 1 (OVOL1) is a key mediator of epithelial lineage determination and mesenchymal–epithelial transition (MET). The cytokines transforming growth factor-β (TGF-β) and bone morphogenetic proteins (BMP) control the epithelial–mesenchymal plasticity (EMP) of cancer cells, but whether this occurs through interplay with OVOL1 is not known. Here, we show that OVOL1 is inversely correlated with the epithelial–mesenchymal transition (EMT) signature, and is an indicator of a favorable prognosis for breast cancer patients. OVOL1 suppresses EMT, migration, extravasation, and early metastatic events of breast cancer cells. Importantly, BMP strongly promotes the expression of OVOL1, which enhances BMP signaling in turn. This positive feedback loop is established through the inhibition of TGF-β receptor signaling by OVOL1. Mechanistically, OVOL1 interacts with and prevents the ubiquitination and degradation of SMAD family member 7 (SMAD7), which is a negative regulator of TGF-β type I receptor stability. Moreover, a small-molecule compound 6-formylindolo(3,2-b)carbazole (FICZ) was identified to activate OVOL1 expression and thereby antagonizing (at least in part) TGF-β-mediated EMT and migration in breast cancer cells. Our results uncover a novel mechanism by which OVOL1 attenuates TGF-β/SMAD signaling and maintains the epithelial identity of breast cancer cells.

## Introduction

Breast cancer is one of the most commonly diagnosed cancers among females worldwide.^1^ Although recent developed therapies have improved the treatment of breast cancer, the survival rate decreases dramatically if patients develop distant metastases.^2^ The epithelial–mesenchymal transition (EMT) process plays a critical role in the invasion–metastasis cascade.^3^ During this process, normal epithelial cells lose cell–cell conjunctions and acquire the fibroblast-like morphology, enhanced migratory and invasive properties.^4,5^ In addition, mesenchymal cancer cells are less sensitive to chemotherapy.^6,7^ At the molecular level, the classic epithelial marker E-cadherin (*ECAD*), which contributes to the cellular adhesion, is transcriptionally repressed by core EMT transcription factors including SNAIL, ZEB and TWIST family members.^8–10^ Moreover, the expression of mesenchymal markers, such as fibronectin (*FN*), N-cadherin (*NCAD*), Vimentin (*VIM*), and α-SMA (*ACTA2*), is upregulated, leading to the cytoskeleton reconstruction and cell migration.^3^ Of note, during the highly dynamic EMT process, a large proportion of cells, which share both epithelial and mesenchymal characteristics, stay in an intermediate and reversible state. The acquisition of this hybrid state is described as epithelial–mesenchymal plasticity (EMP), which is also referred to as partial EMT.^11,12^

The cytokine transforming growth factor-β (TGF-β) is one of the most crucial drivers to induce EMT.^5^ TGF-β initiates cellular responses by specific binding to cell surface TGF-β type II receptor (TβRII) and TGF-β type I receptor (TβRI). Upon TβRII-mediated phosphorylation of TβRI, the latter receptor is activated and induces the phosphorylation and activation of regulatory (R)-SMADs, i.e., SMAD2 and SMAD3. Phosphorylated R-SMADs, upon forming a complex with SMAD4, translocate into the nucleus to activate the expression of typical target genes, such as plasminogen activator inhibitor type 1 (*PAI-1*) and connective tissue growth factor (*CTGF*).^13–15^ Notably, core EMT transcription factors like *SNAIL*, *SLUG*, and *ZEB1*/*2* can be directly induced by the SMAD complex and contribute to TGF-β-mediated changes of aforementioned EMT markers and the reorganization of cytoskeleton such as filamentous (F)-actin.^5^

Bone morphogenetic proteins (BMPs), which are cytokines belonging to the TGF-β family, activate the signaling by inducing complex formation of BMP type II receptor (BMPRII) and BMP type I receptor (BMPRI). Afterward, BMPRI recruits and phosphorylates SMAD1/5/8, which translocate into the nucleus together with SMAD4 and promote the transcription of target genes such as inhibitor of DNA-binding 1 (*ID1*) and inhibitor of DNA-binding 3 (*ID3*).^16^ BMP signaling has been unveiled to maintain epithelial identity and attenuate the metastatic potential of breast cancer cells, which can be achieved by counteracting TGF-β signaling.^17^ Moreover, TGF-β is able to antagonize BMP signaling, indicating the imbalance between TGF-β and BMP may influence the EMT status and invasive abilities of cancer cells.^18–20^

To finely control the propagation of TGF-β signaling, various negative feedback loops and multiple layers of regulation exist.^21^ One of these loops is accomplished by the transcriptional activation of inhibitory (I)-SMAD7. SMAD7 protein is primarily localized in the nucleus, where it interacts with the SMAD-specific E3 ubiquitin protein ligases (SMURFs).^22,23^ In response to TGF-β, the SMAD7/SMURFs complex translocates into the cytoplasm and interacts with TβRI, leading to the proteasome-mediated degradation of TβRI.^22–24^ Furthermore, SMAD7 itself is polyubiquitinated and degraded by several E3 ligases including ARKADIA and RNF12,^25,26^ whereas some deubiquitylating enzymes (DUBs) including USP26 and OTUD1 are capable of removing the polyubiquitin chains off SMAD7.^27,28^

Ovo-like (OVOL) proteins, among which OVOL1 and OVOL2 are better investigated than OVOL3, are pivotal determinants of epithelial lineage and differentiation during embryonic development.^29–32^ Structurally, both OVOL1 and OVOL2 consist of an N-terminal SNAIL1/GFI (SNAG) domain, which acts as a molecular hook to recruit protein partners such as histone deacetylases (HDACs), and four zinc finger domains, which are responsible for the DNA-binding capacity.^32,33^ Due to the structural similarity and identical DNA-binding sequence, OVOL1 and OVOL2 have been reported to function redundantly, e.g., in regulating epithelial plasticity and differentiation of epidermal progenitor cells.^30^ In addition, OVOL1 can suppress the transcription of *OVOL2* and itself.^33,34^

In mesenchymal breast and prostate cancer cells, ectopic expression of OVOL1 or/and OVOL2 can induce mesenchymal–epithelial transition (MET), which is the reverse process of EMT, thereby suppressing cell migration.^35^ Although OVOL2 has been uncovered to attenuate TGF-β-induced EMT in breast cancer cells,^36^ the function of OVOL1 in the regulation of TGF-β/BMP signal transduction during breast cancer progression is ill-defined. Here, we identify *OVOL1* as a potent downstream target of BMP/SMAD and lesser extent of TGF-β/SMAD signaling, whose activities are in turn regulated by OVOL1 in a positive and negative feedback manner, respectively. Furthermore, we elucidate the mechanism by which OVOL1 attenuates TGF-β signaling and breast cancer metastasis. Importantly, OVOL1 interacts with and stabilizes SMAD7. Moreover, 6-formylindolo(3,2-b)carbazole (FICZ) was identified as a compound to potently activate OVOL1 expression, which may offer therapeutic potential for breast cancer patients.

## Results

### *OVOL1* is inversely correlated with EMT and is associated with favorable clinical outcomes in breast cancer patients

**Fig. 1:**
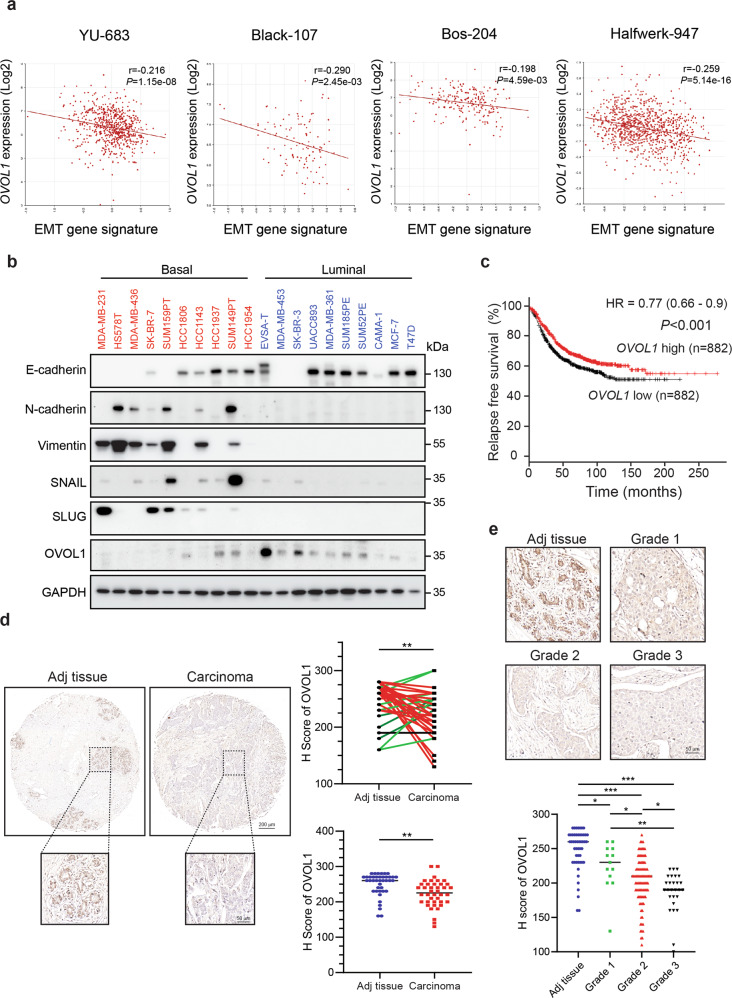
OVOL1 expression is inversely correlated with the EMT gene signature and associated with a favorable prognosis in breast cancer patients. **a** Scatter plot displaying the inverse correlation between *OVOL1* expression and the EMT gene signature in four breast cancer datasets. Titles on top of each panel indicate the datasets in which the RNA-seq data were analyzed. Pearson’s correlation coefficient tests were performed to assess the statistical significance. **b** Western blotting detection of EMT markers and OVOL1 levels in ten basal and ten luminal breast cancer cell lines. GAPDH levels were analyzed to control for equal loading. **c** Kaplan–Meier survival curves illustrating the relapse-free survival of breast cancer patients stratified by *OVOL1* expression. Data were generated from Kaplan–Meier Plotter (https://kmplot.com/analysis/). **d** Representative images of OVOL1 immunohistochemistry results in breast invasive ductal carcinoma (Carcinoma; *n* = 40) and matched cancer adjacent breast tissues (Adj tissue; *n* = 40) (upper). Comparisons between the H score of OVOL1 in the paired tissues (right upper) or unpaired Adj versus Carcinoma tissues (right lower) were performed. Paired tissues with higher OVOL1 expression in Adj tissues compared with Carcinoma are highlighted as red, whereas paired tissues with lower OVOL1 expression in Adj tissues compared with Carcinoma are highlighted as green. The results are expressed as mean ± SD. **0.001 < *P* < 0.01. **e** Representative images of OVOL1 immunohistochemistry staining results in breast invasive carcinoma tissues with different grades (Grade 1, *n* = 13; Grade 2, *n* = 95; Grade 3, *n* = 27) and cancer adjacent breast tissues (Adj tissue; *n* = 50) (upper). Comparison results of OVOL1 H scores in Adj tissues and different groups of carcinoma tissues are shown in the lower panel. The results are expressed as mean ± SD. *0.01 < *P* < 0.05, **0.001 < *P* < 0.01, ***0.0001 < *P* < 0.001

Since OVOL transcriptional repressors have been reported to potentiate MET in prostate and breast cancer cells,^35,36^ we asked whether the expression of three *OVOL* genes was dysregulated during breast cancer progression. A dataset from 51 breast cancer cell lines revealed that *OVOL1* mRNA levels were downregulated in cell lines classified into an aggressive basal subtype, compared with those cell lines grouped into a less aggressive luminal subtype (Supplementary Fig. 1a).^37^ However, no difference of *OVOL2* and *OVOL3* expression between these two subgroups was observed (Supplementary Fig. 1a). This result hints that *OVOL1*, in comparison with the other two *OVOL* members, may play an unique role in breast cancer progression. Since it is pivotal for epithelial cells to acquire the EMP ability to initiate the invasion–metastasis cascade,^4^ we examined the possible association between *OVOL1* expression and an established set of EMT genes in breast cancer patients.^38^ Breast cancer patients with a higher EMT score were considered as more mesenchymal-like, while those with a lower EMT score were considered as more epithelial-like. Interestingly, a significant inverse correlation between *OVOL1* mRNA expression and the EMT signature was observed in four breast cancer patient cohorts (Fig. 1a). Moreover, we found positive correlations between *OVOL1* and epithelial markers (*ECAD*, *EPCAM,* and *KRT18*), and negative correlations between *OVOL1* and mesenchymal markers (*NCAD*, *VIM,* and *FN*), in a breast cancer patient cohort and the aforementioned 51 cell line dataset (Supplementary Fig. 1b, c).^37,39^ E-cadherin and OVOL1 were more prominently expressed, while the mesenchymal markers, including N-cadherin, Vimentin, SNAIL, and SLUG, were lower expressed in ten luminal cell lines than ten basal cell lines (Fig. 1b). In addition, *OVOL1* mRNA and protein levels were determined in commonly used breast cell lines with either epithelial and/or mesenchymal features, including normal breast epithelial cells MCF10A-M1, premalignant breast cells MCF10A-M2 and luminal breast cancer cells MCF7, and highly invasive mesenchymal breast cancer cell lines MDA-MB-436 and MDA-MB-231. Both mesenchymal cell lines with low E-cadherin expression showed very low *OVOL1* mRNA and protein expression (Supplementary Fig. 1d, e). In contrast, *OVOL1* was expressed at high levels in epithelial cell lines, in which E-cadherin was also highly expressed (Supplementary Fig. 1d, e). Notably, survival analysis revealed that breast cancer patients with higher *OVOL1* expression exhibited significantly increased relapse-free survival probabilities than those with lower *OVOL1* expression (Fig. 1c).^40,41^ Moreover, *OVOL1* expression was lower in breast tumor samples than tumor-adjacent normal-like samples (Supplementary Fig. 2a).^39,42^ Additional analysis suggested that *OVOL1* expression was higher in ER-positive (ER+) samples than ER-negative (ER-) samples with poor outcomes (Supplementary Fig. 2b).^39,42^ Subsequently, immunohistochemical staining results demonstrated that OVOL1 levels were reduced in invasive breast ductal carcinoma tissues compared with matched adjacent tissue samples, in 72.5 % (29 of 40) of the paired samples (Fig. 1d). Unpaired analysis also indicated that OVOL1 protein expression was decreased in carcinoma specimens (Fig. 1d). Of note, OVOL1 protein expression was substantially less in samples grouped into more advanced grades (Grade 2 and Grade 3) compared with a more benign grade (Grade 1; Fig. 1e). Taken together, our data suggest that *OVOL1* expression is inversely correlated with EMT and is a potential indicator for a favorable prognosis in breast cancer patients.

### OVOL1 inhibits EMT, migration, extravasation, and early-phase metastatic events in breast cancer cells

**Fig. 2:**
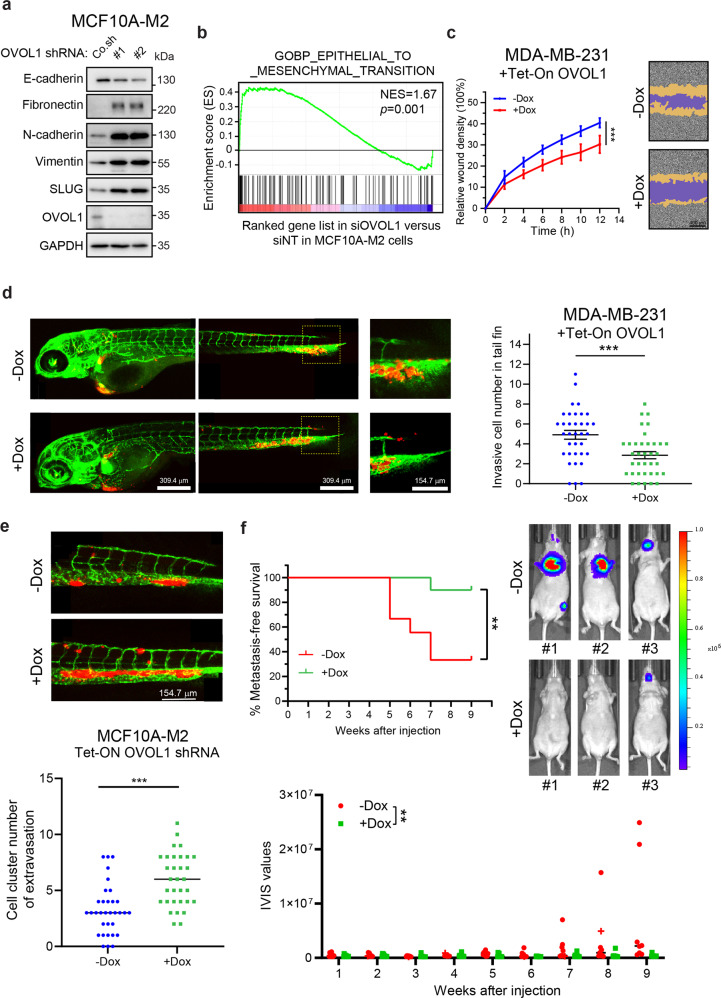
OVOL1 inhibits EMT, migration, extravasation, and early metastatic growth of breast cancer cells. **a** Western blotting quantification of EMT markers expression in MCF10A-M2 cells upon OVOL1 depletion. To control for equal loading, GAPDH levels were analyzed. **b** GSEA analysis of the positive correlation between (manipulated) OVOL1 expression and EMT gene signature. **c** Analysis of real-time migration of MDA-MB-231 cells in the absence or presence of OVOL1 ectopic expression. Cells were either not treated or treated with Doxycycline (Dox) for 2 days prior to seeding. Relative wound density (closure) was plotted at indicated time points (left). Representative scratch wounds are shown at the end time point of the experiment (right). The regions of original scratches and the areas of migrating cells are colored in purple and yellow, respectively. Five biological replicates were included in this assay. The results are expressed as mean ± SD. ***0.0001 < *P* < 0.001. **d** In vivo zebrafish extravasation experiments of MDA-MB-231 cells without or with ectopic expression of OVOL1. MQ water or Dox (to enable induction of OVOL1 expression) was added to egg water from the first day post injection. Representative images with zoom-in of the tail fin area are shown in the left panel. Analysis of the extravasated cell numbers in indicated groups is shown in the right panel. The results are expressed as mean ± SD. ***0.0001 < *P* < 0.001. **e** In vivo zebrafish extravasation experiments of MCF10A-M2 cells without or with the knockdown of OVOL1. MQ water or Dox (to enable the induction of the shRNA targeting OVOL1) was added to the egg water from the first day post injection. Representative images are shown in the upper panel. Analysis of the extravasated cell clusters in indicated groups is shown in the lower panel. The results are expressed as mean ± SD. ***0.0001 < *P* < 0.001. **f** Mouse xenograft cancer model of MDA-MB-231 cells without or with OVOL1 ectopic expression. MQ water or Dox (to enable induction of OVOL1 overexpression) was added to the drinking water from the first day post injection. Metastasis-free survival is depicted in the upper left panel. Log-rank test was used for statistical analysis. **0.001 < *P* < 0.01. Whole-body bioluminescence images (BLI) at 9 weeks of mice are shown in the upper right panel. Analysis of the IVIS values in indicated groups is shown in the lower panel. One mouse in the -Dox group that was terminated at 8 weeks after injection is indicated as a cross (and not taken along in statistical analysis of BLI measurements). The results are expressed as mean ± SD. Two-way ANOVA was used for statistical analysis. **0.001 < *P* < 0.01

Given the inverse correlation between *OVOL1* and EMP, we sought to examine the effect of OVOL1 depletion on EMT in breast cancer cells. Upon OVOL1 knockdown by two independent shRNAs in premalignant MCF10A-M2 breast cells, E-cadherin expression was significantly decreased, while the mesenchymal markers expression, such as fibronectin, N-cadherin, Vimentin and SLUG, was considerably increased (Fig. 2a and Supplementary Fig. 3a). In addition, gene set enrichment analysis (GSEA) demonstrated that OVOL1 depletion was positively correlated with the EMT gene set (Fig. 2b). Moreover, upon OVOL1 knockdown using a doxycycline (Dox)-controlled Tet-On system, epithelial HaCaT keratinocytes obtained mesenchymal-like features as analyzed based on filamentous (F)-actin staining (Supplementary Fig. 3b). The increased cell size upon OVOL1 knockdown was indeed to be expected from cells undergoing EMT. On the contrary, upon the induction of OVOL1 in MDA-MB-231 cells, in which OVOL1 expression was placed under the control of a Dox-induced Tet-On system, epithelial markers (*ECAD*, *EPCAM,* and *KRT18*) expression was enhanced, while mesenchymal markers (*VIM* and *ACTA2*) expression was reduced (Supplementary Fig. 3c). The western blotting analysis also showed that OVOL1 ectopic expression resulted in an increase of E-cadherin expression and a decrease of Vimentin expression in MDA-MB-231 cells (Supplementary Fig. 3d). Wound healing and chemotaxis (transwell migration) assays revealed that OVOL1 ectopic expression led to a decrease of MDA-MB-231 cell migration (Fig. 2c and Supplementary Fig. 3e). Yet, the viability of MDA-MB-231 cells was not affected by OVOL1 overexpression (Supplementary Fig. 3f). Next, mCherry labeled MDA-MB-231 or MCF10A-M2 cells with OVOL1 misexpression were subjected to a zebrafish embryo xenograft model.^43^ Zebrafish fed with Dox that allowed for inducing OVOL1 ectopic expression demonstrated less extravasation of MDA-MB-231 cells into the avascular tail fin area (Fig. 2d). However, MCF10A-M2 cells formed less clusters between blood vessels when zebrafish were fed with Dox to induce OVOL1 knockdown in breast cells (Fig. 2e). Next, we tested the effect of doxycycline-inducible expression of OVOL1 in MDA-MB-231 cells on their metastasis ability after intracardial injection in nude mice. The first MDA-MB-231 cell metastasis was detected much later in the mice that were fed with Dox than without Dox treatment (Fig. 2f). In addition, overexpressing OVOL1 in MDA-MB-231 cells significantly reduced the early metastatic colonization of circulating MDA-MB-231 cells (Fig. 2f and Supplementary Fig. 3g). In conclusion, our results implicate that OVOL1 mitigates the EMT, migration, extravasation, and early-phase metastatic events in breast cancer cells.

### OVOL1 and BMP pathway form a positive feedback loop

**Fig. 3:**
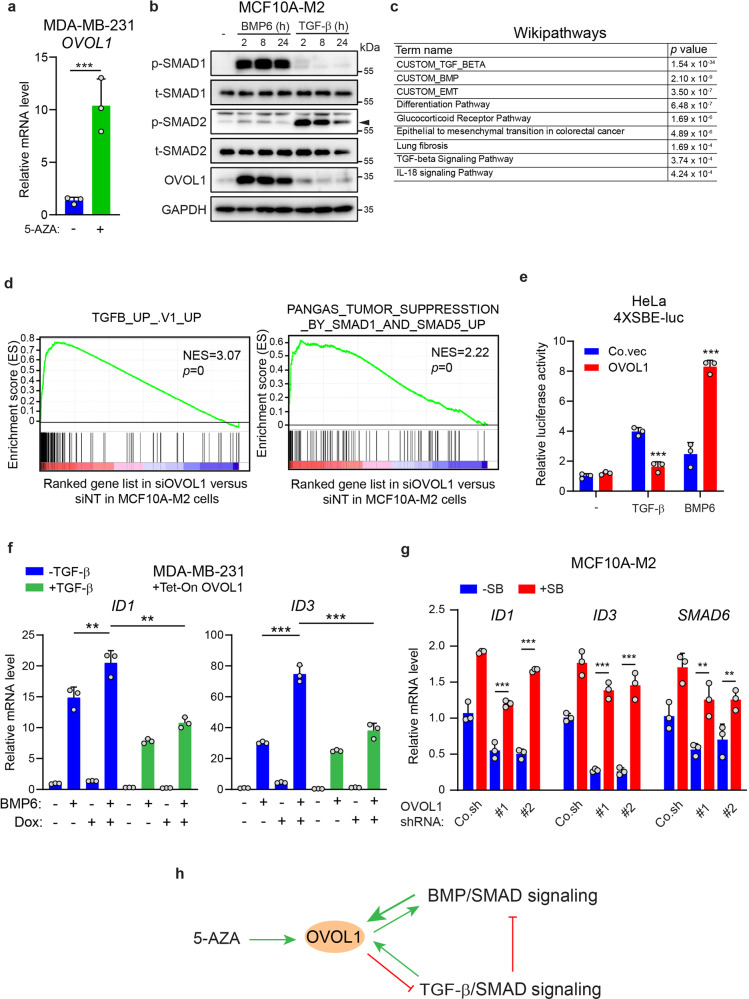
OVOL1 potentiates BMP pathway through mitigating TGF-β signaling. **a** RT-qPCR assay of *OVOL1* expression in MDA-MB-231 cells stimulated without or with 5-Aza-2ʹ-Deoxycytidine (5-AZA) for 7 days. The results are expressed as mean ± SD. ***0.0001 < *P* < 0.001. **b** Western blotting detection of OVOL1 expression in MCF10A-M2 cells treated with TGF-β (5 ng/ml) or BMP6 (50 ng/ml) for indicated time points. The phosphorylation of SMAD1 (p-SMAD1) or SMAD2 (p-SMAD2) was detected to confirm the activation of BMP or TGF-β pathway, respectively. To control for equal loading GAPDH levels were analyzed. **c** The pathway enrichment results from wikipathways upon the depletion of OVOL1 in MCF10A-M2 cells. **d** GSEA analyses of the positive and inverse correlations between (manipulated) OVOL1 expression level and the TGF-β or SMAD1/5 (BMP) gene response signature, respectively. **e** Quantification of the luciferase transcriptional activity in HeLa cells transfected with TGF-β/BMP/SMAD-responsive SBE4-luc reporter and empty vector (Co.vec) or OVOL1. The results are expressed as mean ± SD. ***0.0001 < *P* < 0.001. **f** Detection of *ID1* and *ID3* expression via RT-qPCR in MDA-MB-231 cells with OVOL1 expression induced by Doxycycline (Dox). Cells were treated without or with TGF-β (5 ng/ml) for 1 day followed by Dox stimulation for 2 days. Afterward, cells were serum-starved overnight and stimulated without or with BMP6 (50 ng/ml) for 2 h. The results are expressed as mean ± SD. **0.001 < *P* < 0.01, ***0.0001 < *P* < 0.001. **g** RT-qPCR quantification of *ID1*, *ID3*, and *SMAD6* levels in MDA-MB-231 cells with the inducible expression of OVOL1. Cells were either not treated or treated with SB431542 (SB) for 1 day followed by transduction of empty vector control (Co.sh) or shRNAs targeting OVOL1. The results are expressed as mean ± SD. **0.001 < *P* < 0.01, ***0.0001 < *P* < 0.001. **h** Schematic model illustrating the interplay of BMP and TGF-β pathways with OVOL1

Considering the earlier finding that OVOL1 is expressed at very low to non-detectable levels in basal breast cancer cell lines (Fig. 1b), we speculated that the promoter of *OVOL1* might be epigenetically silenced in these cells. Indeed, RT-qPCR assays showed that 5-Aza-2ʹ-Deoxycytidine (5-AZA), an agent for inducing DNA demethylation, was capable to upregulate *OVOL1* expression in MDA-MB-231 cells (Fig. 3a). Given the finding that *OVOL1* is a target gene of BMP and TGF-β pathways in SMAD4-restored MDA-MB-468 cells and keratinocytes,^44–46^ we tested if *OVOL1* can be induced by BMP and TGF-β in other breast cell lines. In MCF10A-M1 and MCF10A-M2 cells, a strong induction of *OVOL1* expression by BMP6 was observed, whereas TGF-β-mediated induction of *OVOL1* expression was weak (Fig. 3b and Supplementary Fig. 4a, b). In MDA-MB-231 cells, *OVOL1* mRNA could only be moderately induced by TGF-β and BMP6, and a kinetic response upon ligand stimulation was observed (Supplementary Fig. 4c). In addition, treating cells with selective small-molecule kinase inhibitors of BMPR1 (LDN193189; LDN)^47^ or TβR1 type I receptor (SB431542; SB)^48^ efficaciously mitigated the upregulation of *OVOL1* induced by BMP6 or TGF-β (Supplementary Fig. 4d, e). To further determine whether *OVOL1* is a direct target gene of BMP/SMAD and TGF-β/SMAD pathways, we depleted SMAD4 that is essential for the transduction of both pathways.^49,50^ BMP/TGF-β-induced *OVOL1* expression was totally blocked in the absence of SMAD4 (Supplementary Fig. 4f, g). Taken together, our data suggest that *OVOL1* is a target gene of both BMP and TGF-β signaling in breast cancer cells.

Due to the fact that a number of target genes of BMP and TGF-β, such as *SMAD6* and *SMAD7*,^51,52^ can function as modulators to precisely control the pathway transduction, we asked whether OVOL1 misexpression results in the dysregulation of BMP/SMAD or TGF-β/SMAD signaling. To this end, we performed RNA-seq-based transcriptional profiling in MCF10A-M2 cells with OVOL1 depletion (Supplementary Fig. 4h). Pathway enrichment analysis revealed that TGF-β/SMAD and BMP/SMAD pathways were enriched as top pathways modulated by OVOL1 knockdown (Fig. 3c). GSEA analysis confirmed that loss of OVOL1 was positively and negatively correlated with TGF-β and SMAD1/5 (BMP) gene response signatures, respectively (Fig. 3d). Yet, no TGF-β/SMAD or BMP/SMAD pathway enrichment in OVOL2 knockdown cells was revealed by GSEA analysis (Supplementary Fig. 4i, j). As indicated by a TGF-β/BMP/SMAD-driven SBE4-luc reporter,^53^ TGF-β/SMAD signaling was less active, while BMP/SMAD signaling was potentiated, in OVOL1 overexpressing HeLa cells (Fig. 3e). Nevertheless, ectopic expression of OVOL2 to a comparable level as OVOL1 did not regulate the reporter activities as potently as OVOL1 did (Supplementary Fig. 4k, l). In agreement with this result, exposing MDA-MB-231 cells to OVOL1 overexpression significantly augmented the expression of BMP target genes *ID1* and *ID3* (Supplementary Fig. 4m). As TGF-β has been reported to antagonize BMP signaling,^17^ we presumed that the inhibitory effect of OVOL1 on TGF-β signaling might be a reason for the potentiation of BMP signaling. Consistent with this notion, the upregulation of BMP target genes upon OVOL1 ectopic expression was significantly alleviated when cells were pre-challenged with TGF-β (Fig. 3f). In contrast, blocking endogenous TGF-β signaling with a selective TβRI kinase inhibitor (SB431542; SB) rescued the reduction of BMP target genes expression imposed by the absence of OVOL1 (Fig. 3g). Collectively, these results indicate that *OVOL1* is strongly induced by BMP and mildly induced by TGF-β. In turn, OVOL1 has the capacity to augment BMP/SMAD pathway, which is achieved (at least in part) by the inhibition, exerted by OVOL1, on TGF-β/SMAD signaling (Fig. 3h).

### OVOL1 suppresses the TGF-β/SMAD signaling pathway and TGF-β-induced EMT

**Fig. 4:**
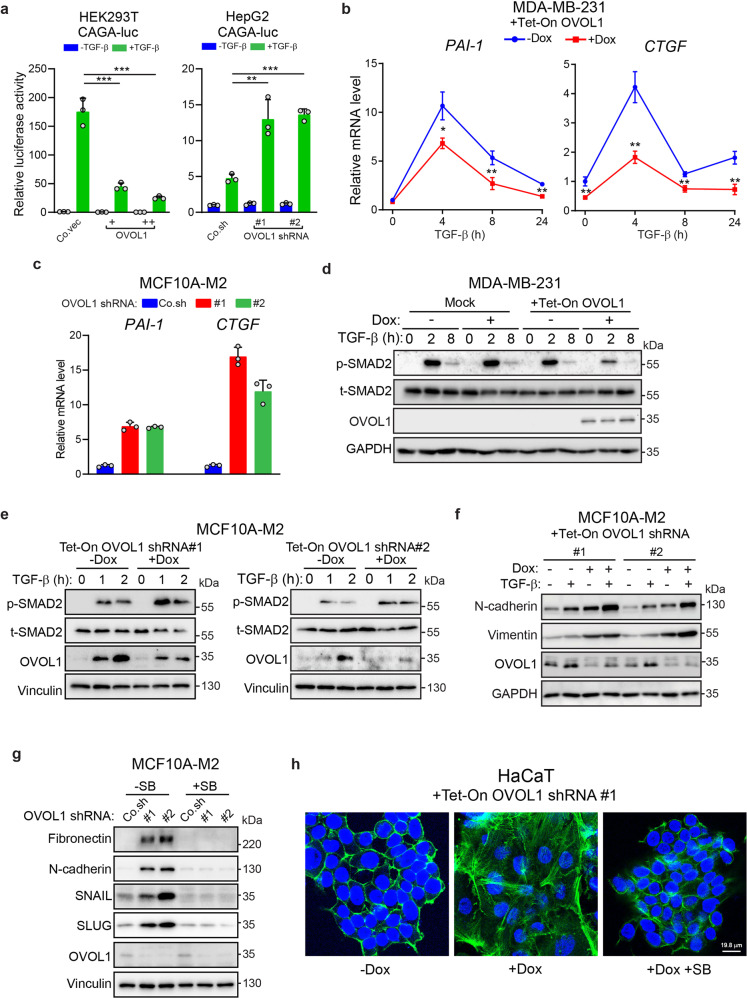
OVOL1 inhibits the TGF-β/SMAD signaling pathway and TGF-β-induced EMT. **a** Reporter assays for measuring the luciferase activity in HEK293T (left panel) or HepG2 cells (right panel) transfected with TGF-β-induced SMAD3/4-dependent CAGA-luc transcriptional reporter and indicated constructs for the ectopic expression (left) or depletion (right) of OVOL1. The results are expressed as mean ± SD. **0.001 < *P* < 0.01, ***0.0001 < *P* < 0.001. **b** *PAI-1* and *CTGF* expression as detected by RT-qPCR in MDA-MB-231 cells with OVOL1 ectopic expression induced by Doxycycline (Dox). Cells were kept in the presence or absence of Dox for 2 days before serum starvation overnight and the treatment of TGF-β (1 ng/ml) for indicated time points. Statistical analyses were carried out at the indicated time points. The results are expressed as mean ± SD. *0.01 < *P* < 0.05, **0.001 < *P* < 0.01. **c** RT-qPCR detection of *PAI-1* and *CTGF* expression in MCF10A-M2 cells upon the knockdown of OVOL1. The results are displayed as mean ± SD in technical triplicates. **d** Western blotting quantification of the phosphorylation of SMAD2 (p-SMAD2) and total SMAD2 (t-SMAD2) in MDA-MB-231 cells without (Mock) or with inducible OVOL1 ectopic expression (+Tet-ON OVOL1). Cells were treated without or with Doxycycline (Dox) for 2 days before serum starvation overnight and the stimulation of TGF-β (1 ng/ml) for indicated time points. To control for equal loading, GAPDH levels were analyzed. **e** The phosphorylation of SMAD2 (p-SMAD2) and total SMAD2 (t-SMAD2) quantified by western blotting in MCF10A-M2 cells with inducible OVOL1 depletion. Cells were kept in the presence or absence of Doxycycline (Dox) for 2 days before the stimulation of TGF-β (1 ng/ml) for indicated time points. **f** Western blotting analysis of changes in mesenchymal markers expression in MCF10A-M2 cells upon OVOL1 knockdown induced by Doxycycline (Dox). Cells were treated without or with Dox for 2 days before the stimulation of TGF-β (2.5 ng/ml) for another 2 days. To control for equal loading, GAPDH levels were analyzed. **g** Western blotting measurement of mesenchymal markers expression in MCF10A-M2 cells with the depletion of OVOL1. Cells were either not treated or treated with SB431542 (SB) for 2 days. To control for equal loading, Vinculin levels were analyzed. **h** Immunofluorescence detection of F-actin and DAPI staining of HaCaT cells upon OVOL1 knockdown induced by Doxycycline (Dox). Cells were treated without or with SB431542 (SB) for 2 h and kept in the presence or absence of Dox for 48 h

We progressed by investigating how OVOL1 mitigates TGF-β signaling transduction. The activity of TGF-β-induced SMAD3/4-driven CAGA-luc transcriptional reporter^15^ was inhibited in OVOL1 overexpressing HEK293T cells, and was potentiated in HepG2 and MCF7 cells with OVOL1 knockdown (Fig. 4a and Supplementary Fig. 5a). These results were further supported in MDA-MB-231 cells in which the expression of classic TGF-β target genes *PAI-1* and *CTGF* was decreased upon OVOL1 overexpression (Fig. 4b). In line with this data, depletion of OVOL1 led to a striking upregulation of TGF-β target genes (Fig. 4c and Supplementary Fig. 5b). Analyses from a breast cancer patient and cell line databases also demonstrated that *OVOL1* expression was inversely correlated with the TGF-β response signature (TBRS)^54^ or the levels of TGF-β/SMAD target genes (Supplementary Fig. 5c–e). Moreover, TGF-β-induced SMAD2 phosphorylation, a read-out of TβRI activity, was suppressed in MDA-MB-231 cells with OVOL1 ectopic expression (Fig. 4d). As a control, TGF-β-induced p-SMAD2 remained unaffected by Dox in parental MDA-MB-231 cells (Mock; Fig. 4d). Similar results were observed in the A549 lung adenocarcinoma cells (Supplementary Fig. 6a). On the contrary, OVOL1 depletion enhanced TGF-β-induced SMAD2 phosphorylation in MCF10A-M2 cells (Fig. 4e). Moreover, TGF-β-triggered N-cadherin and Vimentin protein expression was further potentiated in OVOL1-deficient MCF10-M2 cells, whereas TGF-β-induced changes of EMT markers expression were mitigated in A549 cells upon ectopic expression of OVOL1 (Fig. 4f and Supplementary Fig. 6b). However, pre-treating MCF10A-M2 cells with SB431542 interrupted the upregulation of mesenchymal markers expression caused by the loss of OVOL1, suggesting that the induction of EMT by OVOL1 depletion relies on the potentiation of TGF-β signaling (Fig. 4g and Supplementary Fig. 6c). Finally, immunofluorescence staining of HaCaT cells showed that F-actin rearrangement triggered by the absence OVOL1 could be rescued by SB431542 treatment (Fig. 4h). In conclusion, our results demonstrate that OVOL1 is a negative regulator of the TGF-β/SMAD pathway and TGF-β-driven EMT.

### OVOL1 promotes TβRI degradation by interacting with and stabilizing SMAD7

**Fig. 5:**
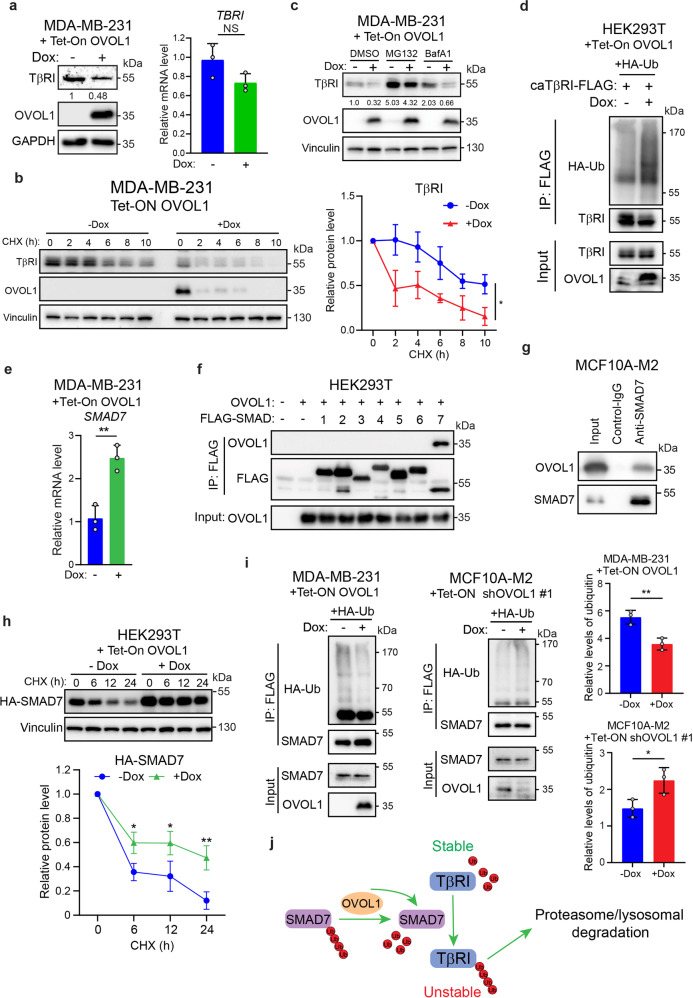
OVOL1 interacts with and stabilizes SMAD7, thereby enhancing the degradation of TβRI. **a** Quantification of TGF-β type I receptor (TβRI) protein (left) or *TBRI* mRNA expression (right) by western blotting or RT-qPCR, respectively, in MDA-MB-231 cells with inducible OVOL1 ectopic expression. Cells were kept in the presence or absence of Doxycycline (Dox) for 2 days. To control for equal loading, GAPDH levels were analyzed. NS not significant. **b** TβRI expression quantified by western blotting in MDA-MB-231 cells with OVOL1 ectopic expression induced by Doxycycline (Dox; left). Cells were cultured in the presence or absence of Dox for 2 days followed by the stimulation of cycloheximide (CHX; 50 µg/ml) for indicated time points. Quantification of the relative protein level of TβRI is shown in the right panel. Statistical analyses were performed at the indicated time points. To control for equal loading Vinculin levels were analyzed. The results are expressed as mean ± SD. *0.01 < *P* < 0.05. **c** Western blotting detection of TβRI expression in MDA-MB-231 cells with the inducible expression of OVOL1. Cells were kept in the presence or absence of Doxycycline (Dox) for 2 days followed by the treatment of proteasome inhibitor MG132 (5 μM) or lysosome inhibitor BafA1 (20 nM) for 6 h. **d** Western blotting quantification of whole-cell lysates (Input) and immunoprecipitants derived from HEK293T cells with inducible OVOL1 expression. Cells were either not stimulated or stimulated with Doxycycline (Dox) for 1 day and then transfected with HA-Ub and constitutively active TβRI-FLAG (caTβRI-FLAG). **e** *SMAD7* expression measured by RT-qPCR in MDA-MB-231 cells with inducible expression of OVOL1. Cells were either not treated or treated with Doxycycline (Dox) for 2 days. The results are expressed as mean ± SD. **0.001 < *P* < 0.01. **f** Western blotting detection of whole-cell lysates (Input) and immunoprecipitants derived from HEK293T cells transfected with indicated FLAG-SMADs and OVOL1. **g** Western blotting quantification of 5% cell lysates (Input) and analysis of OVOL1 and SMAD7 immunoprecipitants derived from MCF10A-M2 cells. The SMAD7 antibody was added to the cell lysates to pull down SMAD7 and the IgG isotype was included as a control. **h** Western blotting measurement of the expression of HA-SMAD7 in HEK293T cells with inducible OVOL1 ectopic expression (upper). Cells were either not treated or treated with Doxycycline (Dox) for 2 days followed by the stimulation of cycloheximide (CHX; 50 ug/ml) for indicated time points. Quantification of the relative protein level of HA-SMAD7 is shown in the lower panel. Statistical analyses were performed at the indicated time points. To control for equal loading Vinculin levels were analyzed. The results are expressed as mean ± SD. *0.01 < *P* < 0.05, **0.001 < *P* < 0.01. **i** Western blotting analysis of whole-cell lysates (Input) and immunoprecipitants derived from MDA-MB-231 (left) or MCF10A-M2 cells (right) stably expressing HA-Ubiquitin (HA-Ub) without or with ectopic expression of OVOL1 (left) or expression the OVOL1 targeting shRNA #1 (middle). Cells were treated without or with Doxycycline (Dox) for 2 days. Total ubiquitination of SMAD7 was probed. Quantification of the relative protein level of HA-Ubiquitin (HA-Ub) is shown in the right panel. The results are expressed as mean ± SD. *0.01 < *P* < 0.05, **0.001 < *P* < 0.01. **j** Schematic working model indicating the action of OVOL1 on SMAD7 and TβRI regulation

Our previous results showed that total SMAD2 levels were not changed by genetically manipulating OVOL1 expression (Fig. 4d, e). This suggested that OVOL1 may modulate TGF-β/SMAD signaling at the receptor level. To test this hypothesis, TβRI expression was evaluated upon OVOL1 misexpression. As shown in Fig. 5a, TβRI protein expression was strongly repressed in MDA-MB-231 cells overexpressing OVOL1, yet *TΒRI* mRNA expression remained the same, indicating that TβRI may be post-translationally inhibited by OVOL1. To investigate TβRI protein stability, a cycloheximide (CHX)-directed protein time-course experiment was carried out. As expected, TβRI showed a shortened half-life in MDA-MB-231 cells subjected to OVOL1 ectopic expression (Fig. 5b). In contrast, depletion of OVOL1 in MCF10A-M2 cells significantly enhanced TβRI stability (Supplementary Fig. 7a). Notably, treating cells with a proteasome inhibitor MG132, but not a lysosome inhibitor BafA1, was able to bypass the OVOL1-directed destabilization of TβRI, suggesting that OVOL1 enhances TβRI degradation via the proteasomal pathway (Fig. 5c). Consistently, the polyubiquitination of TβRI was increased in OVOL1 overexpressing HEK293T cells (Fig. 5d).

Since SMAD7 plays a vital role in the proteasome-mediated ubiquitination of TβRI and OVOL1 is a well-defined transcriptional repressor,^33^ we considered whether *SMAD7* is transcriptionally regulated by OVOL1. Surprisingly, OVOL1 promoted the mRNA expression of *SMAD7* in MDA-MB-231 cells (Fig. 5e). As OVOL1 and SMAD7 were both localized in the nucleus (Supplementary Fig. 7b),^24,33^ we processed by checking whether OVOL1 interacts with SMAD7 protein. Interestingly, OVOL1 was exclusively pulled down by SMAD7, indicating that SMAD7 is a potential binding partner of OVOL1 (Fig. 5f, g and Supplementary Fig. 7c). However, consistent with a previous report,^36^ OVOL2 co-precipitated with SMAD2, SMAD3 and most avidly with SMAD4, and only weakly with SMAD7 (Supplementary Fig. 7d). Next, we asked whether the protein level of SMAD7 is affected due to the interaction with OVOL1. To exclude the impact of transcription, we ectopically expressed SMAD7 in HEK293T cells using an expression plasmid in which expression was driven by heterologous cytomegalovirus (CMV) promoter. Western blotting analysis revealed that the SMAD7 protein expression was enhanced upon OVOL1 ectopic expression, suggesting that OVOL1 may promote SMAD7 protein stability (Supplementary Fig. 7e). This assumption was further validated by a time-course assay demonstrating that the turn-over of SMAD7 protein was significantly mitigated in HEK293T cells overexpressing OVOL1 (Fig. 5h). Furthermore, SMAD7 polyubiquitination was greatly reduced in cells subjected to OVOL1 ectopic expression (Fig. 5i and Supplementary Fig. 7f). On the contrary, SMAD7 polyubiquitination was potentiated in the absence of OVOL1 (Fig. 5i). Given the fact that various ubiquitin chain topologies serve as signals to guide substrates towards different outcomes,^55^ we then analyzed whether OVOL1 impacts the levels of K48 or K63‐incorporated ubiquitin chains on SMAD7. In particular, we identified that the K48 ubiquitin labeled SMAD7, which contributes to protein degradation, was decreased in the presence of OVOL1 overexpression (Supplementary Fig. 7g). However, the K63 ubiquitin level of SMAD7 was not affected by ectopically expressing OVOL1 (Supplementary Fig. 7g). Furthermore, we hypothesized that the interactions between SMAD7 and E3 ligases or DUBs may be altered by OVOL1. Co-immunoprecipitation experiments showed that although interactions between SMAD7 and the reported E3 ligases or DUBs for SMAD7, including ARKADIA, USP26, and OTUD1,^27–29^ were not affected by OVOL1, the E3 ligase RNF12^26^ and SMAD7 interaction was diminished upon the ectopic expression of OVOL1 (Supplementary Fig. 8a). As a consequence, SMAD7 polyubiquitination triggered by RNF12 was partially alleviated when OVOL1 was ectopically expressed (Supplementary Fig. 8b). These results reveal that RNF12 is involved in the decrease of SMAD7 polyubiquitination directed by OVOL1 (Supplementary Fig. 8c). Collectively, our data indicate that OVOL1 upregulates SMAD7 expression at both transcriptional and post-translational levels, which results in the polyubiquitination and degradation of TβRI (Fig. 5j).

### Interaction with SMAD7 is required for the TGF-β signaling mitigation exerted by OVOL1

**Fig. 6:**
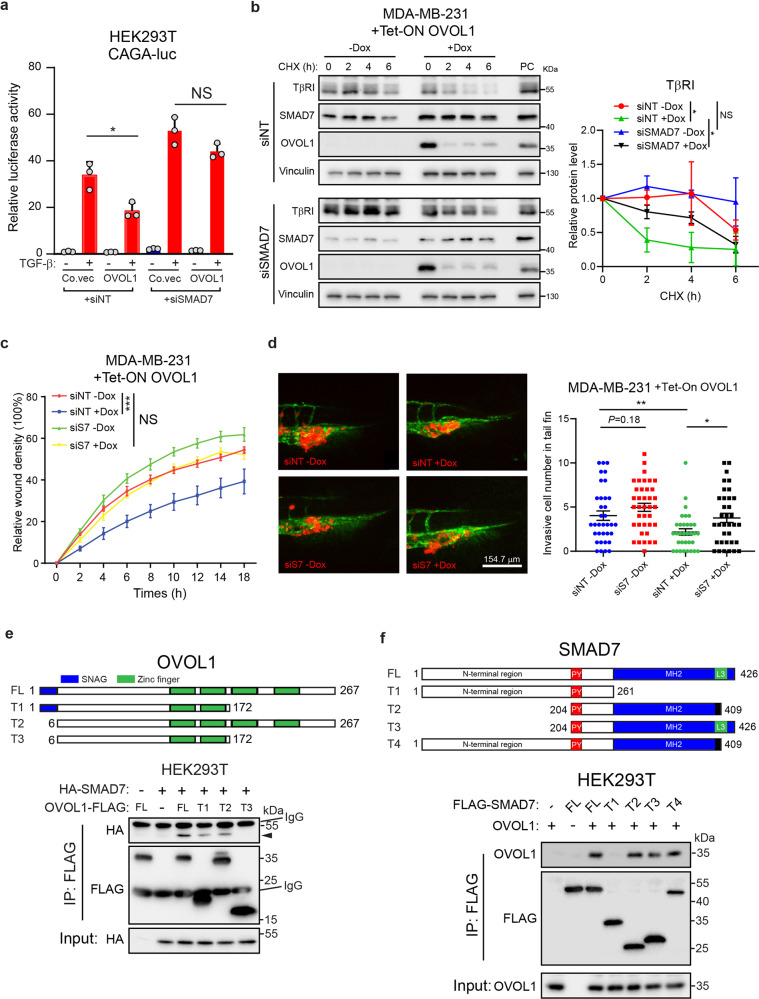
The mitigation of the TGF-β pathway induced by OVOL1 is SMAD7-dependent. **a** Luciferase activity in HEK293T cells transfected with TGF-β-induced SMAD3/4-dependent CAGA-luc transcriptional reporter, OVOL1 and siRNA targeting SMAD7 (siSMAD7). Non-targeting siRNA (siNT) was set as a control for siSMAD7. The results are expressed as mean ± SD. *0.01 < *P* < 0.05. NS not significant. **b** Western blotting detection of TβRI expression in MDA-MB-231 cells with the inducible expression of OVOL1. Cells were transfected with siRNA targeting SMAD7 (siSMAD7) or non-targeting siRNA (siNT) for 1 day. Afterward, cells were kept in the presence or absence of Doxycycline (Dox) for 2 days followed by the stimulation with cycloheximide (CHX; 50 ug/ml) for indicated time points. To control for equal loading Vinculin levels were analyzed. Quantification of the relative protein levels of TβRI is shown in the right panel. The results are expressed as mean ± SD. Statistical analysis by one-way ANOVA was carried out between indicated groups. *0.01 < *P* < 0.05. NS not significant. **c** Real-time scratch-induced migration results of MDA-MB-231 cells without or with ectopic OVOL1 expression. Cells were transfected with siRNA targeting SMAD7 (siS7), followed by the treatment of Doxycycline (Dox) for 2 days prior to seeding. Non-targeting siRNA (siNT) was set as a control for siSMAD7. Relative wound density (closure) was plotted at indicated time points. Three biological replicates were included in this assay. The results are expressed as mean ± SD. ***0.0001 < *P* < 0.001. NS not significant. **d** In vivo zebrafish extravasation assay of MDA-MB-231 cells without or with ectopic OVOL1 expression. Cells were transfected with non-targeting siRNA (siNT) or siRNA targeting SMAD7 (siS7), followed by the injection into zebrafish. MQ water or Doxycycline (Dox) was added to the egg water from the first day post injection. Representative images of the tail fin area are shown in the left panel. Analysis of the extravasated cell numbers in indicated groups is shown in the right panel. The results are expressed as mean ± SD. *0.01 < *P* < 0.05, **0.001 < *P* < 0.01. **e** Schematic representation of the full-length OVOL1 (FL) and truncation mutants (T1–T3) tested (upper). Western blotting analysis of whole-cell lysates (Input) and immunoprecipitants derived from HEK293T cells transfected with HA-SMAD7 and FLAG-tagged full-length OVOL1 or indicated OVOL1 truncation mutants is shown in the lower panel. **f** Schematic representation of full-length SMAD7 (FL) and truncation mutants (T1-T4) tested (upper panel). Western blotting analysis of whole-cell lysates (Input) and immunoprecipitants derived from HEK293T cells transfected with OVOL1 and FLAG-tagged full-length SMAD7 or indicated SMAD7 truncation mutants is shown in the lower panel

Next, we asked whether OVOL1 exerts its inhibitory effect on the TGF-β/SMAD pathway by regulating SMAD7. Consistent with this notion, OVOL1 and SMAD7 suppressed TGF-β/SMAD-driven CAGA-luc transcriptional activity in a synergistic manner (Supplementary Fig. 9a). Conversely, the inhibition of TGF-β/SMAD downstream activity and TβRI stability by OVOL1 overexpression was alleviated upon siRNA-mediated SMAD7 knockdown (Fig. 6a, b and Supplementary Fig. 9b). In addition, OVOL1-induced attenuation of MDA-MB-231 cell migration and extravasation was rescued upon the depletion of SMAD7 (Fig. 6c, d and Supplementary Fig. 9c). Collectively, the inhibitory effects of OVOL1 on TGF-β/SMAD signaling, cell migration, and extravasation are mediated (at least in part) by SMAD7.

To better uncover the mechanism by which OVOL1 interacts with SMAD7, a panel of truncated mutants of OVOL1 protein were generated (Fig. 6e). Co-immunoprecipitation assays showed that the SNAG domain and last two Zinc finger domains of OVOL1 contributed to its optimum interaction with SMAD7 (Fig. 6e). To further assess the functional involvement of SMAD7 in OVOL1-mediated inhibition of TGF-β pathway, MDA-MB-231 cells were exposed to lentivirus bearing constructs that encode full-length (FL) or truncated (T1–T3) OVOL1 proteins, respectively. Interestingly, none of the truncated OVOL1 proteins, whose interactions with SMAD7 were impaired, was capable of suppressing TGF-β-induced SMAD2 phosphorylation, SMAD3/4-driven CAGA-luc transcriptional activity and the induction of TGF-β target genes, as potent as the full-length OVOL1 did (Supplementary Fig. 9d–f). Moreover, the binding regions of OVOL1 on SMAD7 were mapped to further decipher their interaction. We observed that the Mad Homology-2 (MH2) domain-deficient SMAD7 failed to be co-immunoprecipitated together with OVOL1, implying that the MH2 domain of SMAD7 is enrolled in its interaction with OVOL1 (Fig. 6f).

### FICZ induces OVOL1 expression and attenuates the TGF-β pathway, EMT, cell migration, and extravasation

**Fig. 7:**
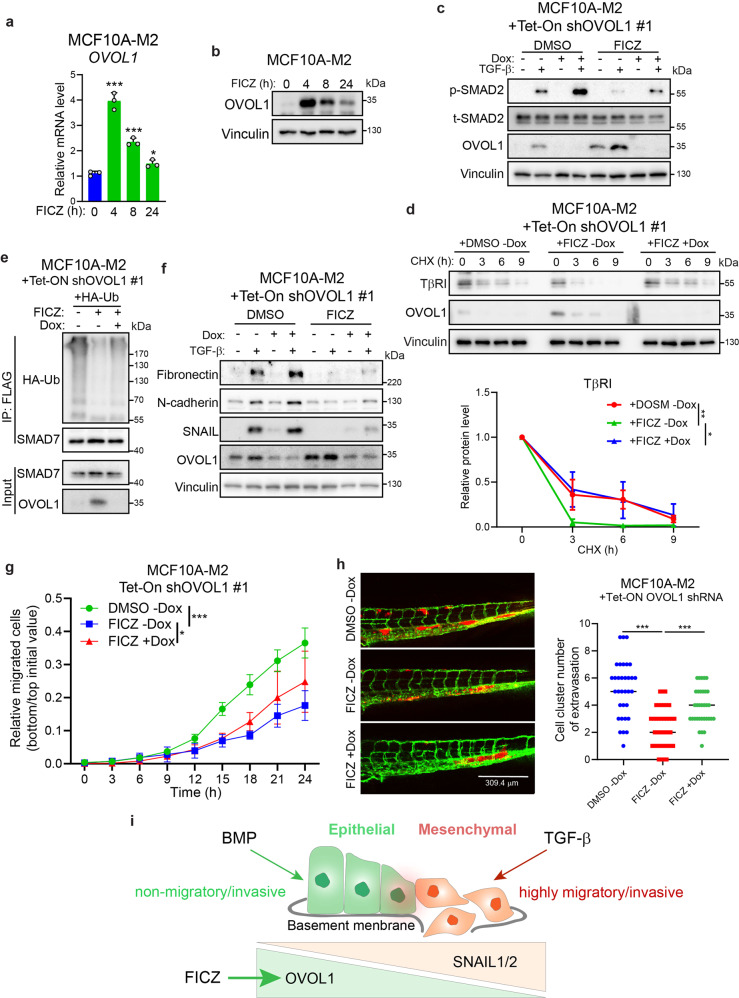
FICZ upregulates OVOL1 expression and mitigates the TGF-β pathway, EMT, cell migration, and extravasation. **a** RT-qPCR detection of *OVOL1* expression in MCF10A-M2 cells stimulated with 6-Formylindolo(3,2-b)carbazole (FICZ; 5 μM) for indicated time points. Statistical analyses were carried out between 0 h group and groups at indicated time points. The results are expressed as mean ± SD. *0.01 < *P* < 0.05, ***0.0001 < *P* < 0.001. **b** Western blotting quantification of OVOL1 expression in MCF10A-M2 cells stimulated with FICZ (5 μM) for indicated time points. Vinculin levels were analyzed to control for equal loading. **c** Western blotting measurement of the phosphorylation of SMAD2 (p-SMAD2) and OVOL1 expression in MCF10A-M2 cells with inducible OVOL1 knockdown by shRNA #1. Cells were stimulated without or with Doxycycline (Dox) for 2 days, followed by FICZ (5 μM) treatment in serum starvation overnight before adding TGF-β (1 ng/ml) for another 2 h. The vehicle control DMSO was included for FICZ. Vinculin levels were analyzed to control for equal loading. **d** Western blotting measurement of the expression of TβRI in MCF10A-M2 cells upon OVOL1 knockdown induced by Doxycycline (Dox). Cells were either not treated or treated with Doxycycline (Dox) for 2 days followed by the stimulation of FICZ (5 μM) overnight. Cycloheximide (CHX; 50 µg/ml) was then added to the medium for indicated time points. Quantification of the relative protein level of TβRI is shown in the lower panel. To control for equal loading Vinculin levels were analyzed. The results are expressed as mean ± SD. Statistical analyses were performed using one-way ANOVA. *0.01 < *P* < 0.05, **0.001 < *P* < 0.01. **e** Western blotting analysis of whole-cell lysates (Input) and immunoprecipitants derived from MCF10A-M2 cells stably expressing HA-Ubiquitin (HA-Ub) without or with expressing the OVOL1 targeting shRNA #1. Cells were treated without or with Doxycycline (Dox) for 2 days and FICZ (5 μM) overnight. Total ubiquitination of SMAD7 was probed. **f** Western blotting analysis of mesenchymal markers expression in MCF10A-M2 cells with inducible OVOL1 knockdown by shRNA #1. Cells were stimulated without or with Doxycycline (Dox) for 2 days, followed by FICZ (5 μM) treatment for 8 h before adding TGF-β (5 ng/ml) for 2 days. The vehicle control DMSO was included for FICZ. **g** IncuCyte real-time chemotaxis assay for evaluating the migration of MCF10A-M2 cells with inducible OVOL1 knockdown by shRNA #1. Cells were pre-treated without or with Doxycycline (Dox) for 2 days before being seeded into the inserts, followed by FICZ (5 μM) treatment. The vehicle control DMSO was included for FICZ. The results are expressed as mean ± SD. *0.01 < *P* < 0.05, *** 0.0001 < *P* < 0.001. **h** In vivo zebrafish embryo xenograft extravasation experiments of MCF10A-M2 cells without or with the knockdown of OVOL1. MQ water or Dox (to enable induction of the shRNA targeting OVOL1) and DMSO or FICZ (1 μM) was added to the egg water from the first day post injection. Representative images are shown in the left panel. Analysis of the extravasated cell clusters in indicated groups is shown in the right panel. The results are expressed as mean ± SD. ***0.0001 < *P* < 0.001. **i** Schematic model of balancing EMP of breast cells by TGF-β, BMP, and downstream transcription factors OVOL1 as well as SNAIL1/2

Since OVOL1 was identified as a suppressor of TGF-β/SMAD signaling and breast cancer progression, we focused on restoring OVOL1 expression by candidate small-molecule compounds. FICZ was reported to promote OVOL1 expression in keratinocytes,^56^ which prompted us to investigate if it is eligible to generate a similar effect in premalignant breast cells. It is noteworthy that FICZ substantially upregulated *OVOL1* mRNA and protein levels in MCF10A-M2 cells (Fig. 7a, b). These results suggested that this drug may be employed to reactivate OVOL1 expression and thereby repressing TGF-β-induced signaling in premalignant cancer cells. Indeed, we observed that TGF-β-induced p-SMAD2, TβRI stability, and SMAD7 polyubiquitination were impinged when MCF10A-M2 cells were stimulated with FICZ (Fig. 7c–e and Supplementary Fig. 10a). However, OVOL1 depletion was able to partially compensate for these inhibitory effects exerted by FICZ, suggesting that OVOL1 may contribute to the suppressive role of FICZ in TGF-β signaling (Fig. 7c–e and Supplementary Fig. 10a). Follow-up experiments showed that FICZ-induced downregulation of mesenchymal markers and inhibition of cell migration and extravasation were mitigated, to some extent, by OVOL1 knockdown (Fig. 7f–h and Supplementary Fig. 10b–d). All these data suggest that restoration of OVOL1 expression by FICZ offers the prospect for therapeutic gain to mitigate overactive pro-oncogenic TGF-β signaling and breast cancer progression.

## Discussion

Our study showed the inhibitory effects of OVOL1 on EMT, cell migration, extravasation, and the early metastatic colonization of breast cells in a mouse cancer xenograft model. We unraveled a novel mechanism by which OVOL1 attenuates TGF-β/SMAD signaling and maintains the epithelial identity of breast cancer cells (Fig. 3h). OVOL1 does so by interacting with and preventing the ubiquitination and degradation of inhibitory SMAD7 (Fig. 5j and Supplementary Fig. 8c). The increased TβRI levels mediate enhanced TGF-β/SMAD signaling and drive breast epithelial cells to transit into cells with more mesenchymal characteristics. In contrast, BMP/SMAD pathway and a small-molecule compound FICZ balance the cell plasticity toward a more epithelial-like status, by (at least in part) the induction of *OVOL1* expression (Fig. 7i).

Despite a variety of evidence from experimental and mathematical modeling analyses characterize OVOL1 as an MET inducer in multiple cancers, the association between OVOL1 and EMP in breast cancer patient remains ill-defined.^35,57–60^ Through analyzing transcriptome datasets, a negative correlation between *OVOL1* and EMT signature was uncovered in various patient cohorts (Fig. 1a). Significantly, we are the first to report that OVOL1 protein is lower expressed in breast invasive carcinoma and is inversely correlated with the progression of breast cancer towards aggressive grades, which demonstrates that the absence of OVOL1 expression may be expected to aid in detecting aggressive breast cancers (Fig. 1d, e).

The initiation of cancer metastasis involves in the augmented migratory and invasive capacities that are conferred by EMT on primary tumor cells.^3^ Upon extravasating into the parenchyma of distant organs, it seems that cancer cells undergo MET to support the outgrowth of micrometastases.^4,61^ We have clearly shown that OVOL1 suppresses EMT, cell migration, in vivo extravasation using a zebrafish embryo xenograft model, and early-stage metastatic colonization using a mouse xenograft model (Fig. 2a–f). Of note, we found that ectopic expression of OVOL1 does not affect MDA-MB-231 cell proliferation/viability (Supplementary Fig. 3f). Yet, we cannot exclude the possibility that OVOL1-mediated MET may potentiate the outgrowth of micrometastases at the last step of cancer metastasis in vivo. Therefore, further investigation is required to comprehensively examine the role of OVOL1 in the invasion–metastasis cascade.^4^

One of the key molecular hallmarks of EMT is the absence of E-cadherin expression. In highly invasive breast cancer cells including MDA-MB-231, *ECAD* promoter is hypermethylated, resulting in the loss of E-cadherin expression.^62^ *ECAD* expression was shown to be dramatically enhanced in MDA-MB-231 cells ectopically expressing OVOL1, which is consistent with the data shown by Roca and colleagues,^35^ indicating that OVOL1 may be engaged in regulating the methylation of *ECAD* promoter (Supplementary Fig. 3c, d). Thus it is worth to further investigate whether OVOL1 can work together with some demethylating enzymes to alleviate the methylation status of *ECAD* promoter. Moreover, the possibility that OVOL1 may antagonize the effects of particular methylating enzymes on *ECAD* promoter cannot be excluded.

*OVOL2* promoter is hypermethylated in late-stage colorectal cancer patients.^63^ We observed that 5-AZA dramatically upregulated *OVOL1* mRNA expression in MDA-MB-231 cells (Fig. 3a). To determine whether this 5-AZA effect is direct or indirect, further evidence on the methylation status within the *OVOL1* promoter is needed. It has been reported that OVOL1 functions downstream of WNT signaling in differentiating epidermal cells and hair follicles.^64^ Here, we have identified *OVOL1* as a target gene of BMP/SMAD and TGF-β/SMAD pathways (Fig. 3b). This result is in agreement with the previous report that *OVOL1* expression can be induced by TGF-β in keratinocytes^45,46^ or by TGF-β and BMP in MDA-MB-468 cells with SMAD4 restoration,^44^ although no biological relevance was investigated in these studies. In this study, we found OVOL1 as a potentiator of BMP signaling, which is, at least partially, achieved by the attenuation of TGF-β signaling (Fig. 3f–h). Similarly, OVOL1 was reported to enhance osteoblast differentiation by activating *BMP2* transcription.^65^ In contrast, the transcription of a typical BMP target gene *ID1* can be directly suppressed by OVOL1 during trophoblasts development.^66^ These findings indicate that the mechanism by which OVOL1 modulates BMP signaling may be context-dependent.

Given the pivotal role in mediating the cross-talk between TGF-β pathway and many other signaling pathways, SMAD7 itself is under strict control by multiple mechanisms and layers of regulation.^24^ As a target gene of the TGF-β/SMAD pathway and thereby mediating important negative feedback control, transcription of *SMAD7* is activated by the SMAD complex together with co-regulators.^24^ Our results indicate that OVOL1 is capable to promote SMAD7 expression at both transcriptional and post-translational levels (Fig. 5). Interestingly, although ectopic expression of OVOL1 in MDA-MB-231 cells significantly decreases the TGF-β downstream reporter activity and the expression of TGF-β target genes, i.e., *PAI-1* and *CTGF*, the *SMAD7* mRNA is strikingly increased (Fig. 5e). OVOL1 was initially identified as, and generally functions as, a transcriptional repressor by binding to the CCGTTA element within the promoter of target genes.^33^ The transcriptional repressor SNAIL, which also contains the N-terminal SNAG domain as OVOL proteins do, was shown to function as a transcriptional activator by binding to specific enhancer regions.^67^ Although the canonical binding element of OVOL1 cannot be found in the promoter region of SMAD7 (data not shown), we cannot exclude the possibility that, upon forming a complex with other protein partners such as transcriptional activators, OVOL1 may bind to and potentiate the activity of some enhancer regions, thereby enhancing the transcription of a particular panel of genes such as *SMAD7*. Moreover, it is also likely that OVOL1 regulates the transcription of *SMAD7* in an indirect manner.

At the post-translational level, SMAD7 is targeted for polyubiquitination and degradation by E3 ligases such as ARKADIA and RNF12,^25,26^ whose effects can be counteracted by DUBs like USP26 and OTUD1.^27,28^ OVOL1 was observed to decrease K48 ubiquitin of SMAD7 and thereby enhancing SMAD7 protein stability (Fig. 5h–i and Supplementary Fig. 7f, g). We hypothesized that OVOL1 may act as a scaffold to bring DUBs to SMAD7 or impair interactions between E3 ligases and SMAD7. Mechanistically, we showed that RNF12 and SMAD7 interact, and that the resulting polyubiquitination of SMAD7, were attenuated by OVOL1 ectopic expression (Supplementary Fig. 8a–c). However, we cannot rule out the possibility that other proteins (DUBs or E3 ligases) may also participate in OVOL1-mediated deubiquitylation of SMAD7. To further investigate (other potential) underlying mechanisms, proteomic interactome analysis for OVOL1 can be performed to identify novel and relevant interacting E3 ligases or DUBs.

In a previous study, ectopic expression of OVOL2 was observed to inhibit TGF-β/SMAD signaling in NMuMG cells through directly interacting with SMAD4 and interfering in the complex formation between SMAD2/3 and SMAD4.^36^ Results from our SMAD-driven transcriptional (SBE) reporter assays indicated that the induction of TGF-β/SMAD signaling and BMP/SMAD signaling are more potently affected upon the ectopic expression of OVOL1 than OVOL2 (Fig. 3e and Supplementary Fig. 4k, l). Moreover, RNA-seq-based profiling of transcriptional changes and GSEA upon OVOL2 knockdown demonstrated that TGF-β/SMAD and BMP/SMAD signaling pathways are not affected (Supplementary Fig. 4i, j). OVOL1 strongly interacts with SMAD7 but not with the other SMAD proteins. OVOL2, however, interacts with SMAD2, SMAD3, and most avidly with SMAD4, but only weakly interacts with SMAD7 (Supplementary Fig. 7d). These latter results indicate that despite the structural similarity, and in some cases functional redundancy,^32^ these two OVOL proteins contribute to the regulation of TGF-β/SMAD (and BMP/SMAD) signaling via diverse mechanisms.

TGF-β pathway is hyperactivated during breast cancer progression, making it an attractive therapeutic target.^68^ We have validated the inhibitory role of OVOL1 on TGF-β pathway transduction and TGF-β-induced EMT (Fig. 4), suggesting that restoration of OVOL1 expression may be an option for targeting the pro-oncogenic TGF-β signaling in breast cancer.

We have identified FICZ as an activator to trigger OVOL1 expression and thereby suppressing TGF-β/SMAD signaling, EMT, migration and extravasation of breast cancer cells. These latter results may provide opportunities to mitigate breast cancer progression (Fig. 7). Although depletion of OVOL1 rescues, to some extent, the inhibitory effects of FICZ, other targets of FICZ may also contribute to its suppressive role in breast cancer progression. Along those lines, FICZ was reported to induce the expression of tumor-suppressing microRNAs *miR-22*, *miR-515-5p*, and *miR-124-3p* in MCF7 breast cancer cells.^69^ Moreover, the mammosphere formation ability of MCF7 cells is inhibited by FICZ,^70^ indicating FICZ may function as a suppressor for breast cancer cell malignancy. FICZ is an UV-derived tryptophan photoproduct whose inhibitory effects have been shown in inflammatory diseases such as chronic mite-induced dermatitis.^71^ It will be interesting to explore the potential of FICZ in the treatment of breast cancer in (pre)clinical models. We report here that FICZ inhibits breast cancer extravasation in zebrafish xenografts, a model from which we previously showed that the results can be validated in mouse xenograft cancer metastasis models.^17,72–74^ Although adult mice with FICZ treatment in relatively high concentrations do not have pathological signs,^75,76^ systematic toxicity studies in animal models are required to evaluate the clinical potential of FICZ. Moreover, OVOL1 agonists with higher specificity and in vivo safety can be explored to enable therapeutic gain for breast cancer patients.

## Materials and methods

### Cell culture and reagents

HEK293T, HeLa, HepG2, A549, MDA-MB-231, and MCF7 were purchased from American Type Culture Collection (ATCC) and SUM149PT cells were obtained from Dr. Sylvia Le Dévédec (Leiden Academic Center for Drug Research, Leiden, the Netherlands). All the cell lines were cultured in Dulbecco’s modified Eagle medium (DMEM; Thermo Fisher Scientific; Cat. No.: 41965062) supplemented with 10% fetal bovine serum (FBS; Thermo Fisher Scientific; Cat. No.: 16000044) and 100 U/ml penicillin/streptomycin (Thermo Fisher Scientific; Cat. No.: 15140163). MCF10A-Ras (MCF10A-M2) cells were derived from MCF10A cells transformed with Ha-Ras and were kindly provided by Dr. Fred Miller (Barbara Ann Karmanos Cancer Institute, Detroit, USA). MCF10A and MCF10A-M2 cells were maintained in DMEM/F12 (GlutaMAX™ Supplement; Thermo Fisher Scientific; Cat. No.: 31331028) containing 5% horse serum (Thermo Fisher Scientific; Cat. No.: 26050088), 0.1 μg/ml Cholera toxin (Sigma-Aldrich; Cat. No.: C8052), 0.02 μg/ml Epidermal Growth Factor (EGF; Sigma-Aldrich; Cat. No.: 01-107), 0.5 μg/ml Hydrocortisone (Sigma-Aldrich; Cat. No.: H0135), 10 μg/ml Insulin (Sigma-Aldrich; Cat. No.: I6634) and 100 U/ml penicillin/streptomycin. All the cell lines mentioned were maintained in a 5% CO_2_, 37 °C, humidified incubator and tested negative for mycoplasma routinely. Human cell lines were checked for authenticity by short tandem repeats (STR) profiling. The 20 human breast cancer cell lines used for detecting OVOL1 (and epithelial and mesenchymal markers) expression were described previously.^77^ Doxycycline (Dox; Sigma-Aldrich; Cat. No.: D9891) or 5-Aza-2ʹ-Deoxycytidine (5-AZA; Sigma-Aldrich; Cat. No.: 189826) was added to the culture medium at a final concentration of 100 ng/ml and 5 μM, respectively. 6-Formylindolo(3,2-b)carbazole (FICZ; Sigma-Aldrich; Cat. No.: SML1489) dissolved in DMSO were used at 5 μM. Lysosome inhibitor BafA1 (Sigma-Aldrich; Cat. No.: B1793) was used to treat cells at 20 nM final concentration. Selective small-molecule kinase inhibitors of BMPR1 (LDN193189; LDN)^47^ and TβR1 type I receptor (SB431542; SB)^48^ were used at a concentration of 120 nM and 5 μM, respectively. Recombinant BMP6 and TGF-β3 were a kind gift from Slobodan Vukicevic (University of Zagreb) and Andrew Hinck (University of Pittsburgh), respectively.

### Generation of constructs

Human *OVOL1* cDNA was amplified by PCR from MCF10A-M2 cells and subcloned into the lentiviral vector pLV-bc-CMV-puro. The inducible vector for OVOL1 ectopic expression was generated using Gateway cloning into the pLIX-403 vector (Addgene; Cat. No.: 41395). We used two shRNAs TRCN0000015679 (#1) and TRCN0000015681 (#2) from Sigma MISSION® shRNA library for OVOL1 knockdown. The sequences targeting OVOL1 AGTGTCACAACGACGTCAAGA (#1) and AGGATTTGATGGCTACCAAAT (#2) were cloned into the lentiviral FH1tUTG vector to generate lentiviral constructs for inducible OVOL1 knockdown.

### Lentiviral transduction and transfections

Third-generation lentiviral packaging vectors (VSV, gag, and Rev) and cDNA or shRNA expressing constructs were transfected into HEK293T cells. Cell supernatants were collected at 48 h post transfection. To generate stable cell lines, cells were plated at 10% confluence and infected by lentiviral supernatants supplemented with the same volume of fresh medium and 8 ng/ml Polybrene (Sigma-Aldrich; Cat. No.: 107689) for 24 h. After 48 h of infection, cells were selected with Puromycine (1 μg/ml; Sigma-Aldrich; Cat. No.: P9620) for 3 days. For transfection of non-targeting siRNA (Dharmacon), siRNA SMARTpool targeting *SMAD7* (Dharmacon; Cat. No.: L-020068-00-0005), *OVOL1* (Dharmacon; Cat. Nr.: L-006543-01-0005) or *OVOL2* (Dharmacon; Cat. No.: L-013793-02-0005), cells were seeded at 80% confluence and incubated with complex formed by DharmaFECT transfection reagents and siRNA (10 nM at final concentration). Medium was changed at 24 h post transfection. RNA samples were collected 2 days after transfection.

### Real-time quantitative PCR (RT-qPCR)

RNA was extracted by a NucleoSpin RNA kit (Macherey Nagel; Cat. No.: 740955) according to the manufacturer’s instructions. Subsequently, reverse transcription was performed using a RevertAid RT Reverse Transcription Kit (Thermo Fisher Scientific; Cat. No.: K1691). Indicated genes were detected by specific primer pairs on the generated cDNA using the CFX Connect Real-Time PCR Detection System (Bio-Rad). *GAPDH* was used as a reference transcript. The results are expressed as mean ± SD, *n* = 3. 2^−ΔΔCt^ method was applied to analyze the relative expression. Primer sequences used in this study are listed in Supplementary Table 1.

### Western blotting

Cells were lysed with RIPA buffer (150 mM sodium chloride, 1.0% Triton X-100, 0.5% sodium deoxycholate, 0.1% SDS and 50 mM Tris-HCl, pH 8.0) containing freshly added complete protease inhibitor cocktail (Roche; Cat. No.: 11836153001). Protein concentrations were measured by a DC™ protein assay kit (Bio-Rad; Cat. No.: 5000111) according to the manufacturer’s instructions. Equal amounts of proteins were loaded and separated by Sodium Dodecyl Sulfate polyacrylamide gel electrophoresis (SDS-PAGE). Afterward, proteins were transferred onto a 0.45-μm polyvinylidene difluoride (PVDF) membrane (Merck Millipore; Cat. Nr.: IPVH00010). Subsequently, 5% non-fat dry milk dissolved in Tris-buffered saline (TBS) with 0.1% Tween 20 (TBST) was used to block the membrane for 1 h at room temperature (RT). Membranes were probed with the respective primary and secondary antibodies. Clarity™ Western ECL Substrate (Bio-Rad; Cat. No.: 1705060) and ChemiDoc Imaging System (Bio-Rad; Cat. No.: 17001402) were used to detect the signal. Primary antibodies used in this study are listed in Supplementary Table 2. Secondary antibodies used in this study are anti-IgG (Sigma-Aldrich; Cat. No.: NA931V) and anti-rabbit (Cell Signaling; Cat. No.: 7074 S). All results were derived from at least three independent biological replicates, and representative results are shown. Protein levels were quantified by densitometry using ImageJ (National Institutes of Health, USA).

### Co-immunoprecipitation assays

HEK293T or MCF10A-M2 cells were lysed with TNE lysis buffer (50 mM Tris-HCl, pH 7.4, 1 mM EDTA, 150 mM NaCl, and 1% NP40) containing freshly added complete protease inhibitor cocktail and kept on ice for 15 min. The lysates were centrifuged at 1.4 × 10^4^×*g* for 10 min at 4 °C. Equal amounts of protein were incubated with anti-FLAG agarose beads (Sigma-Aldrich; Cat. No.: A2220) for 30 min at 4 °C with rotation. For checking the interaction between endogenous SMAD7 and OVOL1, 1 µL antibodies against SMAD7 (R&D; Cat. No.: MAB2029) were added to the cell lysates and the mixtures were kept overnight at 4 °C with rotation. The next day, 20 µL Pierce™ Protein G Agarose (Thermo Fisher; Cat. No.: 20397) were added and kept for 2 h at 4 °C with rotation. Beads were washed five times with the TNE buffer for 5 min at 4 °C with rotation. Afterward, samples were boiled with 2× sample buffer for 5 min and subjected to SDS-PAGE analysis. Primary antibodies used for western blotting are listed in Supplementary Table 2.

### CAGA-luc or SBE-luc transcriptional reporter assays

HEK293T, HeLa, or HepG2 cells were seeded in the wells of a 24-well plate (Corning) at a density of 3 × 10^5^ and were transfected with 100 ng SMAD3/4-driven transcriptional CAGA-luc^15^ or BMP/TGF-β/SMAD-responsive SBE4-luc reporter,^53^ 80 ng β-galactosidase encoding plasmids and 320 ng indicated constructs using polyethyleneimine (PEI). For co-transfection of siRNAs and plasmids, HEK293T were seeded in the wells of a 24-well plate (Corning) at a density of 3 × 10^5^ and transfected with 20 ng SMAD3/4-driven transcriptional CAGA-luc reporter, 50 ng β-galactosidase encoding plasmids, 430 ng expression construct encoding OVOL1 or empty vector control, and non-targeting siRNA or siRNA SMARTpool targeting *SMAD7* (10 nM) using the DharmaFECT 1 transfection reagent (Horizon; Cat. No.: T-2001). At 16 h after transfection, cells were serum-starved for 8 h and then kept in the presence or absence of TGF-β3 (1 ng/ml) or BMP6 (10 ng/ml) overnight. Luciferase activity was measured using D-luciferin (Promega) as a substrate with a luminometer (PerkinElmer). The relative luciferase reporter activity was normalized to the β-galactosidase activity. All the experiments were repeated at least three times in biologically independent experiments, and representative results are shown.

### MTS cell proliferation assays

MTS assay was carried out to evaluate the cell viability followed with the manufacturer’s instructions (Promega; Cat. No.: G3581). Cells were seeded at a density of 1 × 10^3^ cells in wells of 96-well plates (Corning). The absorbance of the samples was measured at 490 nm with a luminometer at 1, 2, 3, 4, and 5 days after seeding. Six biological replicates were included in each group.

### Immunofluorescence staining

HeLa cells were transfected with constructs encoding TβRI-FLAG, OVOL1 or EGFP-SMAD7. Two days post transfection, 4% paraformaldehyde (PFA) was used for fixing cells on covering glass for 20 min at RT, after which cells were permeabilized by phosphate-buffered saline (PBS) supplemented with 0.1% Triton X for 10 min. Afterward, non-specific binding was blocked with 3% bovine serum albumin (BSA) dissolved in PBS for 1 h at RT. For staining of filamentous (F)-actin, cells were incubated with the Alexa Fluor 488 Phalloidin (Thermo Fisher Scientific; Cat. No.: A12379) in 1:500 dilution for 30 min at RT. To determine the localization of TβRI, OVOL1, and SMAD7, cells were incubated with primary antibodies in 1:100 dilution for 1 h at RT. After three times of washing with PBS, the specimens were probed with secondary antibodies (Invitrogen; Cat. No.: A21428 and A28175) in a dilution of 1:1000 for 1 h at RT. The specimens were then subjected to three washes with PBS and mounted with VECTASHIELD antifade mounting medium with DAPI (Vector Laboratories; Cat. No.: H-1200). The images were captured using a Leica SP8 confocal microscope (Leica Microsystems) and analyzed with the aid of LAS X software.

### Ubiquitination assays

HEK293T cells transfected with indicated constructs or MDA-MB-231 + Tet-ON OVOL1 cells and MCF10A-M2 + Tet-ON shOVOL1 #1 cells stimulated with Dox for 2 days were treated 5 h prior to harvesting with 5 µM proteasome inhibitor MG132 (Sigma-Aldrich; Cat. No.: 474787). After washing with cold PBS twice containing 10 mM N-ethylmaleimide (NEM; Sigma-Aldrich; Cat. Nr.: E3876), cells were lysed in 1% SDS-RIPA buffer (25 mM Tris-HCl, pH 7.4, 150 mM NaCl, 1% NP40, 0.5% sodium deoxycholate and 1% SDS) consisting of protease inhibitors and 10 mM NEM. Lysates were subsequently boiled for 5 min and diluted to 0.1% SDS at a final concentration in RIPA buffer. Then protein concentrations were measured and the same amount of proteins were incubated with anti-FLAG agarose beads for 30 min at 4 °C. For checking the ubiquitination of endogenous SMAD7, 1 µL antibodies against SMAD7 were added to cell lysates and the mixtures were kept overnight at 4 °C with rotation. The next day, 20 µL Pierce™ Protein G Agarose (Thermo Fisher; Cat. No.: 20397) was added and kept for 2 h at 4 °C with rotation. After five times of washing with RIPA buffer, beads were boiled in 2× loading buffer for 5 min and separated by SDS-PAGE. All the experiments were repeated at least three times in biologically independent experiments, and representative results are shown.

### IncuCyte wound-healing migration assays

Cells were seeded at a density of 5 × 10^4^ in the wells of 96-well Essen ImageLock plate (Essen BioScience; Cat. No.: 4379). After 16 h, cells were serum-starved with DMEM medium supplemented with 0.5% FBS for 8 h. The scratch wounds were generated by the Wound Maker (Essen BioScience). Floating cells were discarded by washing with PBS and attached cells were incubated with DMEM medium supplemented with 0.5% FBS. Migration was monitored in the IncuCyte live-cell imaging system (Essen BioScience). Relative wound density was analyzed by the IncuCyte cell migration software. All the experiments were repeated at least three times in biologically independent experiments, and representative results are shown as mean ± SD.

### IncuCyte chemotaxis cell migration assay

In brief, 40 μl MCF10A-M2 or SUM149PT cells suspended in medium supplemented with 0.5% serum were seeded at a density of 1 × 10^3^ in the inserts of an Incucyte clearview 96-well plate (Essen BioScience; Cat. No.: 4582). 20 μl medium supplemented with 0.5% serum-containing indicated compounds or corresponding vehicle controls was added into the inserts. Afterward, cells were allowed to settle at ambient temperature for 20 min. In parallel, 200 μl medium supplemented with 10% serum was added to the reservoir plates. Then the inserts containing cells were placed into a pre-filled plate. Cells on the top and bottom of inserts were imaged and analyzed using the IncuCyte system. The experiments were repeated two times, and representative results are shown as mean ± SD.

### Statistical analyses

Statistical analysis was performed using Graphpad Prism 7 software. Results were expressed as the mean ± SD of triplicates. All measurements were taken from distinct samples. For analysis, unpaired Student’s *t* test was used and *P* < 0.05 was considered to be statistically significant. Paired Student’s *t* test was carried out for analyzing the statistical significance of matched tissue samples in Fig. 1d. Two-way analysis of variance (ANOVA) was applied for statistical analysis of real-time migration and chemotaxis assays. Pearsons’ coefficient tests were performed to assess statistical significance for correlations between the expression of two genes or gene signatures.

## Supplementary information

SUPPLEMENTAL MATERIAL

## Data Availability

The RNA-seq data presented in the study are deposited in the GEO repository, and the accession number in GEO is GSE192548.
