# Supplementary material for: OVOL1 inhibits breast cancer cell invasion by enhancing the degradation of TGF-β type I receptor
Source: Signal Transduct Target Ther. 2022 Apr 29;7:126. doi: 10.1038/s41392-022-00944-w (PMC9050647; doi:10.1038/s41392-022-00944-w)
Supplement: Supplementary file 1 — SUPPLEMENTAL MATERIAL [file 41392_2022_944_MOESM1_ESM.docx]

Supplementary Materials for

**OVOL1 inhibits breast cancer cell invasion by enhancing the degradation of TGF-β type I receptor**

Chuannan Fan^1,2^, Qian Wang^1^, Gerard van der Zon^1,2^, Jiang Ren^1^, Cedrick Agaser^3^, Roderick C Slieker^1,4^, Prasanna Vasudevan Iyengar^1,2^, Hailiang Mei^3^, and Peter ten Dijke^1,2*^

1 Department of Cell and Chemical Biology, Leiden University Medical Center, 2300 RC, Leiden, The Netherlands.

2 Oncode Institute, Leiden University Medical Center, Leiden, 2300 RC, The Netherlands.

3 Department of Biomedical Data Sciences, Sequencing Analysis Support Core, Leiden University Medical Center, 2300 RC, Leiden, The Netherlands.

4 Department of Epidemiology and Data Science, Amsterdam Public Health Institute, Amsterdam Cardiovascular Sciences Institute, Amsterdam UMC, location VUmc, 1081 HV, Amsterdam, the Netherlands.

Correspondence to: p.ten_dijke@lumc.nl

**This PDF file includes:**

Materials and Methods

Figures. S1 to S10

Tables S1 and S2

**Materials and Methods**

**Data analyses of gene expression in clinical patient samples and breast cancer cell lines**

Four publicly available gene expression datasets (GSE102484^1^, GSE36771^2^, GSE12276^3^ and GSE3494^4^) derived from the R2 Genomics Analysis and Visualization Platform (R2 platform; <http://r2.amc.nl>) were applied for the analyses of the correlations between *OVOL1* mRNA levels and the EMT or TGF-β response gene signature. Using the GSE12777 dataset^5^, *OVOL1*, *OVOL2* or *OVOL3* expression in 51 breast cell lines was compared. In the same dataset, correlations between *OVOL1* and EMT markers or TGF-β target genes expression were carried out. Differential expression data of *OVOL1* mRNA level in TCGA patients were derived from the MOBCdb database^6^. Correlations between *OVOL1* and the TGF-β target genes were evaluated in 1097 TCGA patients from R2 platform^7^. Pearson’s correlation coefficient tests were performed to assess the statistical significance.

**RNA-seq-based transcriptional profiling, pathway enrichment and GSEA analysis**

Two days post-transfection, cells were collected for RNA samples preparation by the NucleoSpin RNA kit. After mRNA enrichment by Oliogo dT selection and the following library preparation, RNA-seq was performed in the DNBseq platform (Beijing Genomics Institute, BGI, Hongkong). Afterwards, RNA-Seq files were processed using the opensource BIOWDL RNAseq pipeline v5.0.0 (https://zenodo.org/record/5109461#.Ya2yLFPMJhE) developed at the LUMC. This pipeline performs FASTQ preprocessing (including quality control, quality trimming, and adapter clipping), RNA-Seq alignment, read quantification, and optionally transcript assembly. FastQC was used for checking raw read quality. Adapter clipping was performed using Cutadapt (v2.10) with default settings. The alignment of RNA-Seq reads was carried out using STAR (v2.7.5a) on GRCh38 human reference genome. The gene read quantification was conducted using HTSeq-count (v0.12.4) with setting “–stranded=no”. The gene annotation used for quantification was Ensembl version 104. Using the gene read count matrix, counts per million mapped reads (CPM) was calculated per sample on all annotated genes. Genes with a higher CPM than 1 in at least 25% of all samples are kept for downstream analysis. This provided us with 11574 genes for the analysis between siOVOL1 and siNT group, and 11565 genes for the analysis between siOVOL2 and siNT group. For the differential gene expression analysis, dgeAnalysis R-shiny application (https://github.com/LUMC/dgeAnalysis/tree/v1.4.4) was used. EdgeR (v3.34.1) with trimmed mean of m-values (TMM) normalization was used to perform differential gene expression analysis. Benjamini and Hochberg false discovery rate (FDR) was computed to adjust p-values obtained for each differentially expressed gene. Using a cutoff of 0.05 at the adjust p-values, we identified all up and down regulated genes. Using the differentially expressed genes as inputs, the pathway enrichment analysis was then performed with the aid of wikipathways symbol in gProfiler R package. Gene set enrichment analysis (GSEA) was carried out using the GSEA software^8, 9^. TGF-β (TGFB_UP.V1_UP) gene response signature^10^, SMAD1/5 (BMP) (PANGAS_TUMOR_SUPPRESSION_BY_SMAD1_AND_SMAD5_UP) gene response signature^11^ and EMT (GOBP_EPITHELIAL_TO_MESENCHYMAL_

TRANSITION; GO: 0001837) gene signature were set as references to determine the correlations between (manipulated) OVOL1 or OVOL2 expression and TGF-β/SMAD signaling, BMP/SMAD signaling or EMT, respectively.

**Immunohistochemical (IHC) staining and evaluation**

Tissue microarray slides consisting of cancer adjacent breast tissues and matched breast carcinoma tissues (Biomax; Cat. Nr.: BR804b), and breast invasive carcinoma tissues with different grades (Biomax; Cat. Nr.: BC081116d) were dried overnight at 37°C. The next day, paraffine was removed by placing slides in xylene thrice, followed by placing in 100% ethanol twice. Endogenous peroxidase activity was blocked by 0.3% hydrogen peroxide for 20 min. Tissue sections were rehydrated in 96%, 70% and 50% Ethanol, respectively. Subsequently, antigen retrieval (10 min boiling in 0.01 M Sodium Citrate, pH 6.0) was performed after washing slides with PBS with 0.1% Tween 20 (PBST) for 5 min. Slides were cooled down to RT, followed by 3 times washing with PBST. Primary antibody against OVOL1 (Thermo Fisher; Cat. Nr.: PA5-41480) diluted (1:200) in 1% BSA (dissolved in PBST) was applied to incubate slides overnight at 4°C. Slides were washed with PBST for 3 times before incubating with 1: 200 diluted biotinylated secondary antibody (DAKO; Cat. Nr.: E0353) for 30 min at RT. After 3 times washing with PBST, slides were subjected to Vectastain complex (Vector Laboratories; Cat. Nr.: PK-6100) incubation for 30 min at RT. Afterwards, slides were washed with PBST thrice and developed by DAB. Next, slides were counterstained with Mayers Haematoxylin (Sigma-Aldrich; Cat. Nr.: MHS80) for 10 s and dehydrated. Finally, Entellan (Merck; Cat. Nr.: 107961) was applied to mount the slides. Images were captured by slide scanner (3D Histech Pannoramic 250). Staining was quantified and expressed as a H score which was determined by the formula 3 × the percentage of strongly staining cells + 2 × the percentage of moderately staining cells + the percentage of weakly staining cells.

**Zebrafish extravasation assay of human breast cancer cells**

Transgenic zebrafish lines Tg (fli1: EGFP) were raised according to standard procedures in compliance with the local Institutional Committee for Animal Welfare of the Leiden University. Zebrafish extravasation assays were performed as previously described^12^. Zebrafish were washed with PBS twice and fixed with 4% PFA at 5 days after injection. Imaging of the zebrafish were carried out with the aid of an inverted SP5 STED confocal microscope (Leica). At least thirty zebrafish were analyzed for each group and representative images were taken. All the experiments were repeated for 2 times, and representative results are shown.

**Mice xenograft model**

22 five week-old female BALB/c nu/nu mice were ordered and acclimatized for one week in the Laboratory Animal Center (LAC) of the Netherlands Cancer Institute (Amsterdam, The Netherlands). All the mice were anesthetized by the inhalation of isoflurane (0.8 L/min) and intracardially injected with MDA-MB-231 luc^+^ Tet-ON OVOL1 cells (3 × 10^5^ /100 μl PBS) through the left heart ventricle. Three mice died after one day post-injection and the rest 19 mice were subdivided into two groups (9 in the -Dox groups and 10 in the +Dox group). Mice in the +Dox group were fed with 1% sucrose water supplemented with 2 mg/ml Doxycycline (Sigma; Cat. Nr.: D9891) to induce OVOL1 expression in MDA-MB-231 cells, while mice in the -Dox group were fed with the 1% sucrose water as a vehicle control. Bioluminescence imaging was carried out once a week to monitor the growth of metastases. All the mice experiment procedures were approved by the Animal Welfare Committee of the Netherlands Cancer Institute (Amsterdam, The Netherlands).

**References**

1. Cheng, S.H.C. et al. Validation of the 18-gene classifier as a prognostic biomarker of distant metastasis in breast cancer. *PloS One* **12**, e0184372 (2017).

2. Caldon, C.E. et al. Cyclin E2 Overexpression Is Associated with Endocrine Resistance but not Insensitivity to CDK2 Inhibition in Human Breast Cancer Cells. *Mol. Cancer Ther.* **11**, 1488-1499 (2012).

3. Bos, P.D. et al. Genes that mediate breast cancer metastasis to the brain. *Nature* **459**, 1005-1009 (2009).

4. Miller, L.D. et al. An expression signature for p53 status in human breast cancer predicts mutation status, transcriptional effects, and patient survival. *Proc. Natl. Acad. Sci. U. S. A.* **102**, 13550-13555 (2005).

5. Hoeflich, K.P. et al. In vivo Antitumor Activity of MEK and Phosphatidylinositol 3-Kinase Inhibitors in Basal-Like Breast Cancer Models. *Clin. Cancer Res.* **15**, 4649-4664 (2009).

6. Xie, B.B. et al. MOBCdb: a comprehensive database integrating multi-omics data on breast cancer for precision medicine. *Breast Cancer Res. Treat.* **169**, 625-632 (2018).

7. Cancer Genome Atlas Network. Comprehensive molecular portraits of human breast tumours. *Nature* **490**, 61-70 (2012).

8. Subramanian, A. et al. Gene set enrichment analysis: a knowledge-based approach for interpreting

genome-wide expression profiles. *Proc. Natl. Acad. Sci. U. S. A.* **102**, 15545-15550 (2005).

9. Mootha, V.K. et al. PGC-1-alpha-responsive genes involved in oxidative phosphorylation are coordinately downregulated in human diabetes. *Nat. Genet.* **34**, 267-273 (2003).

10. Padua, D. et al. TGFbeta primes breast tumors for lung metastasis seeding through angiopoietin-like 4. *Cell* **133**, 66-77 (2008).

11. Pangas, S.A. et al. Conditional deletion of Smad1 and Smad5 in somatic cells of male and female gonads leads to metastatic tumor development in mice. *Mol. Cell Biol.* **28**, 248-257 (2008).

12. Ren, J., Liu, S., Cui, C. & ten Dijke, P. Invasive behavior of human breast cancer cells in embryonic zebrafish. *J. Vis. Exp.* **122**, e55459 (2017).


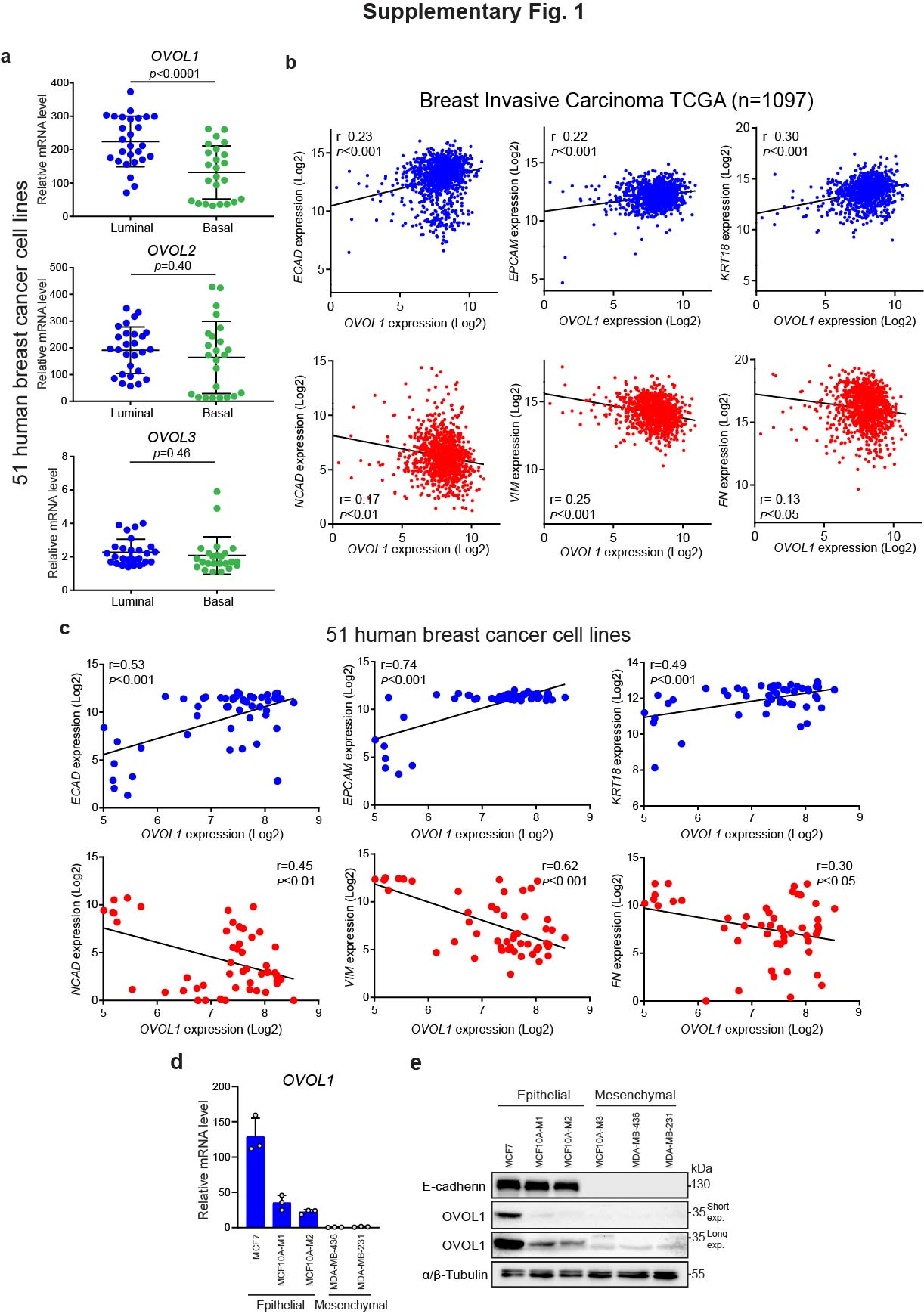


**Supplementary Fig. 1 *OVOL1* expression is positively correlated with the expression of epithelial markers while negatively correlated with the expression of mesenchymal markers.**

(**a**) *OVOL1*, *OVOL2* or *OVOL3* mRNA expression in 51 breast cell lines that were subdivided into luminal and basal groups. (**b**, **c**) Scatter plots demonstrating positive correlation between the expression of *OVOL1* and epithelial markers (*ECAD*, *EPCAM* or *KRT18*) or inverse correlation with mesenchymal markers (*NCAD*, *VIM* or *FN*) in datasets consisting of TCGA breast invasive carcinoma samples (**b**) or 51 human breast cancer cell lines (**c**). (**d**) RT-qPCR analysis of *OVOL1* expression in normal breast cells (MCF10A-M1), pre-malignant breast cells (MCF10A-M2) and luminal breast cancer cells (MCF7) with an epithelial phenotype and triple negative breast cancer cell lines with an aggressive mesenchymal phenotype (MDA-MB-436 and MDA-MB-231). The result is presented as mean ± SD in technical triplicates. (**e**)Western blot analysis of E-cadherin and OVOL1 protein levels in normal breast cells (MCF10A-M1), pre-malignant breast cells (MCF10A-M2) and luminal breast cancer cells (MCF7) with an epithelial phenotype, and triple negative breast cancer cell lines with an aggressive mesenchymal phenotype (MDA-MB-436 and MDA-MB-231). α/β-tubulin levels were analyzed to control for equal loading.


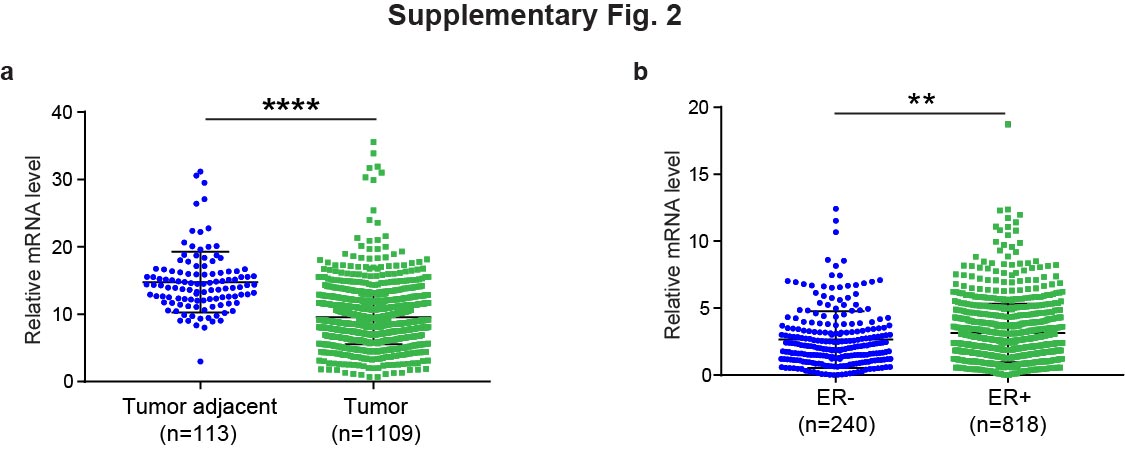


**Supplementary Fig. 2 *OVOL1* is lower expressed in tumor samples with poor prognosis.**

(**a**) Differential *OVOL1* mRNA expression in tumor adjacent normal breast tissue samples and tumor samples from breast cancer patients. (**b**) Differential analysis of *OVOL1* mRNA expression in breast cancer patient specimens that were subdivided into ER negative (ER-) and ER positive (ER+) groups. The results in (**a**) and (**b**) are expressed as mean ± SD.  ** 0.001 <  *p* < 0.01, **** *p* < 0.0001.


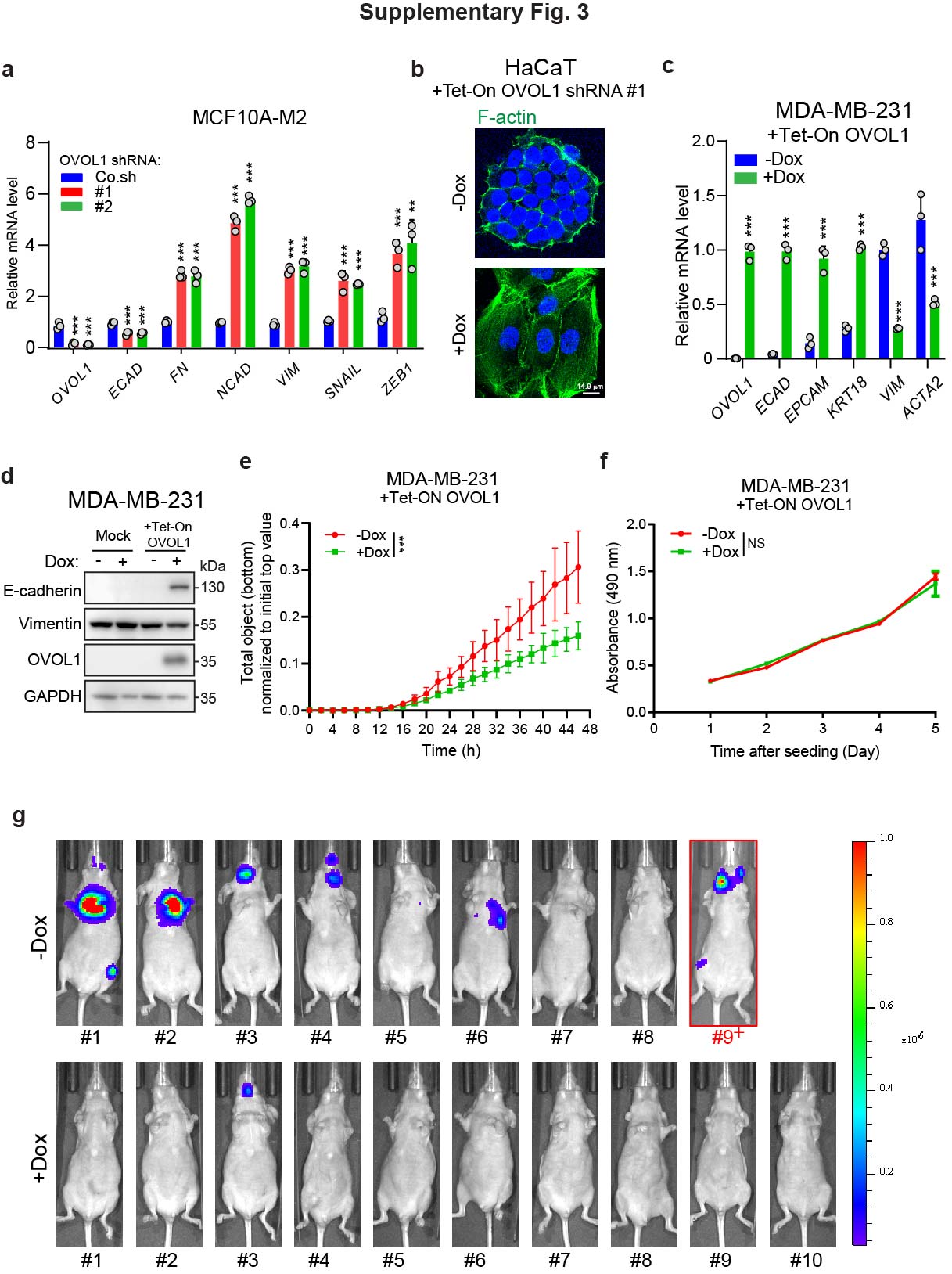


**Supplementary Fig. 3 OVOL1 inhibits the EMT and MDA-MB-231 breast cancer cell migration and metastasis but not MDA-MB-231 cell viability.**

(**a**) Measurement of EMT markers expression by RT-qPCR in MCF10A-M2 cells upon OVOL1 knockdown. Statistical analyses were performed between groups of vector control (Co.sh) and OVOL1 sh#1 or sh#2, respectively. The results are expressed as mean ± SD. ** 0.001 <  *p* < 0.01, *** 0.0001 < *p* < 0.001. (**b**) Immunofluorescence detection of F-actin and 4, 6-diamidino-2-phenylindole (DAPI) staining of HaCaT cells upon OVOL1 knockdown induced by Doxycycline (Dox). Cells were treated without or with Dox for 48 h. Effectivity of *OVOL1* gene knockdown was confirmed (**Supplementary Fig. 3a**). Scale bar, 14.9 μM. (**c**) RT-qPCR detection of EMT markers expression in MDA-MB-231 cells in the absence or presence of OVOL1 ectopic expression. Cells were either not stimulated or stimulated with Doxycycline (Dox) for 2 days. Statistical analyses were carried out between -Dox and +Dox groups. The results are expressed as mean ± SD. *** 0.0001 < *p* < 0.001. (**d**) Analysis expression of EMT markers by western blotting in MDA-MB-231 cells without (Mock) or with inducible OVOL1 expression (+Tet-On OVOL1). Cells were treated without or with Doxycycline (Dox) for 2 days. To control for equal loading GAPDH levels were analyzed. (**e**) IncuCyte chemotaxis assay to evaluate the migratory abilities of MDA-MB-231 cells upon the ectopic expression of OVOL1. Cells were stimulated without or with Doxycycline (Dox) two days before being seeded in the chambers. The results are expressed as mean ± SD. *** 0.0001 < *p* < 0.001, NS, not significant. (**f**) MTS cell viability analysis of MDA-MB-231 cells without or with ectopic expression of OVOL1. Cells were kept in the presence or absence of Doxycycline (Dox) for indicated time points. See **Supplementary Fig. 3c** for the analysis of *OVOL1* expression after Dox challenge. (**g**) Whole body bioluminescence images (BLI) of mice treated without or with Dox. Images of all but one mice at 9 weeks are shown; one mouse without Dox treatment was imaged at 8 weeks and thereafter terminated. Three selected mice without and with Dox are shown in **Fig. 2f**.


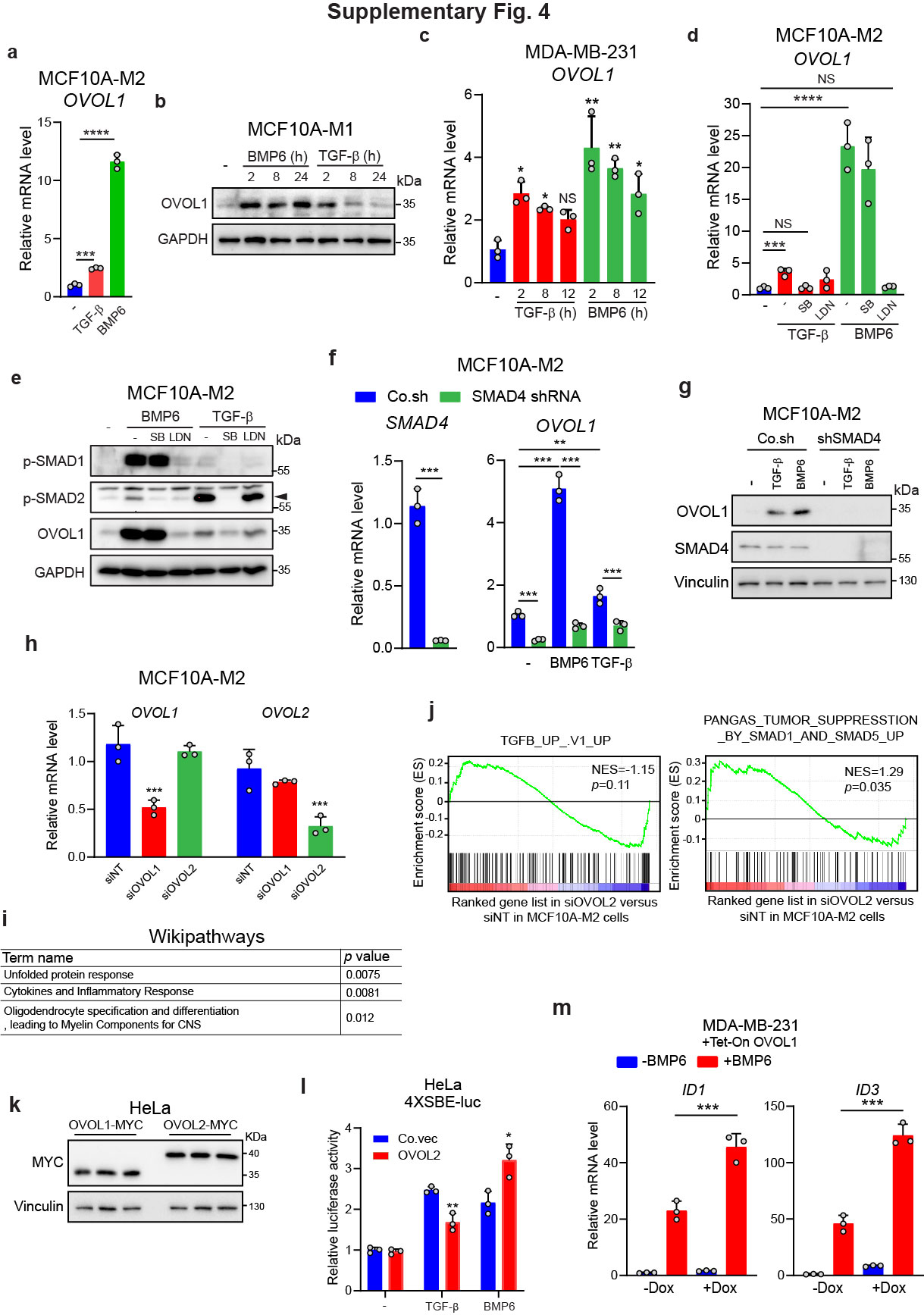
 **Supplementary Fig. 4 *OVOL1* is a target gene of BMP/SMAD and TGF-β/SMAD pathways.**

(**a**) *OVOL1* expression detected by RT-qPCR in MCF10A-M2 cells upon the stimulation of TGF-β (5 ng/ml) or BMP6 (50 ng/ml) for indicated time points. Statistical analyses were performed between control group and indicated groups with ligand treatment. The results are expressed as mean ± SD.  *** 0.0001 < *p* < 0.001, **** *p* < 0.0001. (**b**) Western blotting detection of OVOL1 expression in MCF10A cells treated with vehicle control (-) or TGF-β (5 ng/ml) or BMP6 (50 ng/ml) for indicated time points. To control for equal loading GAPDH levels were analyzed. (**c**) *OVOL1* expression detected by RT-qPCR in MDA-MB-231 cells upon the stimulation of TGF-β (5 ng/ml) or BMP6 (50 ng/ml) for indicated time points. Statistical analyses were performed between control group and indicated groups with ligand treatment. The results are expressed as mean ± SD. * 0.01 < *p* < 0.05, ** 0.001 < *p* < 0.01, NS, not significant. (**d**) *OVOL1* expression quantified by RT-qPCR in MCF10A-M2 cells. Cell were either not treated or treated with small molecule kinase inhibitors of BMP type I receptor (LDN193189; LDN; 120 nM) or TGF-β type I receptor (SB431542; SB; 5 μM) for 30 min followed by the stimulation of TGF-β (5 ng/ml) or BMP6 (50 ng/ml) for 2 h. The results are expressed as mean ± SD. *** 0.0001 < *p* < 0.001, **** *p* < 0.0001, NS, not significant. (**e**) Western blot analysis of OVOL1 protein expression in MCF10A-M2 cells. Cells were kept in the presence or absence of inhibitors of BMP (LDN193189; LDN) or TGF-β (SB431542; SB) for 30 min followed by the treatment of vehicle control (-), TGF-β (5 ng/ml) or BMP6 (50 ng/ml) for 4 h. The phosphorylation of SMAD1 (p-SMAD1) or SMAD2 (p-SMAD2) was detected to confirm the activation of the BMP or TGF-β pathway, respectively. To control for equal loading GAPDH levels were analyzed. (**f**) *OVOL1* expression quantification by RT-qPCR in MCF10A-M2 cells upon SMAD4 depletion. Cells were serum starved overnight and treated with vehicle control (-), TGF-β (5 ng/ml) or BMP6 (50 ng/ml) for 2 h. The results are expressed as mean ± SD. ** 0.001 < *p* < 0.01, *** 0.0001 < *p* < 0.001. (**g**) Western blotting assay for detecting OVOL1 expression in MCF10A-M2 cells without (Co.sh) or with (shSMAD4) SMAD4 depletion. Cells were serum starved overnight and treated with vehicle control (-), TGF-β (5 ng/ml) or BMP6 (50 ng/ml) for 4 h. To control for equal loading Vinculin levels were analyzed. (**h**) RT-qPCR evaluation of *OVOL1* or *OVOL2* levels in MCF10A-M2 cells transfected with the indicated siRNAs. Comparisons were performed against the siNT group. The results are expressed as mean ± SD. *** 0.0001 < *p* < 0.001. (**i**) The pathway enrichment results from wikipathways when OVOL2 was depleted in MCF10A-M2 cells. (**j**) GSEA analyses of the correlations between (manipulated) OVOL2 expression level and the TGF-β (left) or SMAD1/5 (BMP; right) gene response signature. (**k**) Western blotting quantification for OVOL1 and OVOL2 expression in HeLa cells transfected with MYC-tagged OVOL1 or OVOL2. Cell lysates in three biological replicates from the luciferase assays in **Fig. 3e** and **Supplementary Fig. 4l** were analyzed. To control for equal loading Vinculin levels were analyzed. (**l**) Quantification of the luciferase transcriptional activity in HeLa cells transfected with BMP/TGF-β-responsive SBE4-luc reporter and empty vector (Co.vec) or OVOL2. The results are expressed as mean ± SD. * 0.01 < *p* < 0.05, ** 0.001 < *p* < 0.001. (**m**) Measurement of *ID1* and *ID3* expression by RT-qPCR in MDA-MB-231 cells with inducible OVOL1 expression. Cells were either not treated or treated with Doxycycline (Dox) for 2 days before serum starvation overnight and adding BMP6 (50 ng/ml) for 2 h. The results are expressed as mean ± SD. *** 0.0001 < *p* < 0.001.


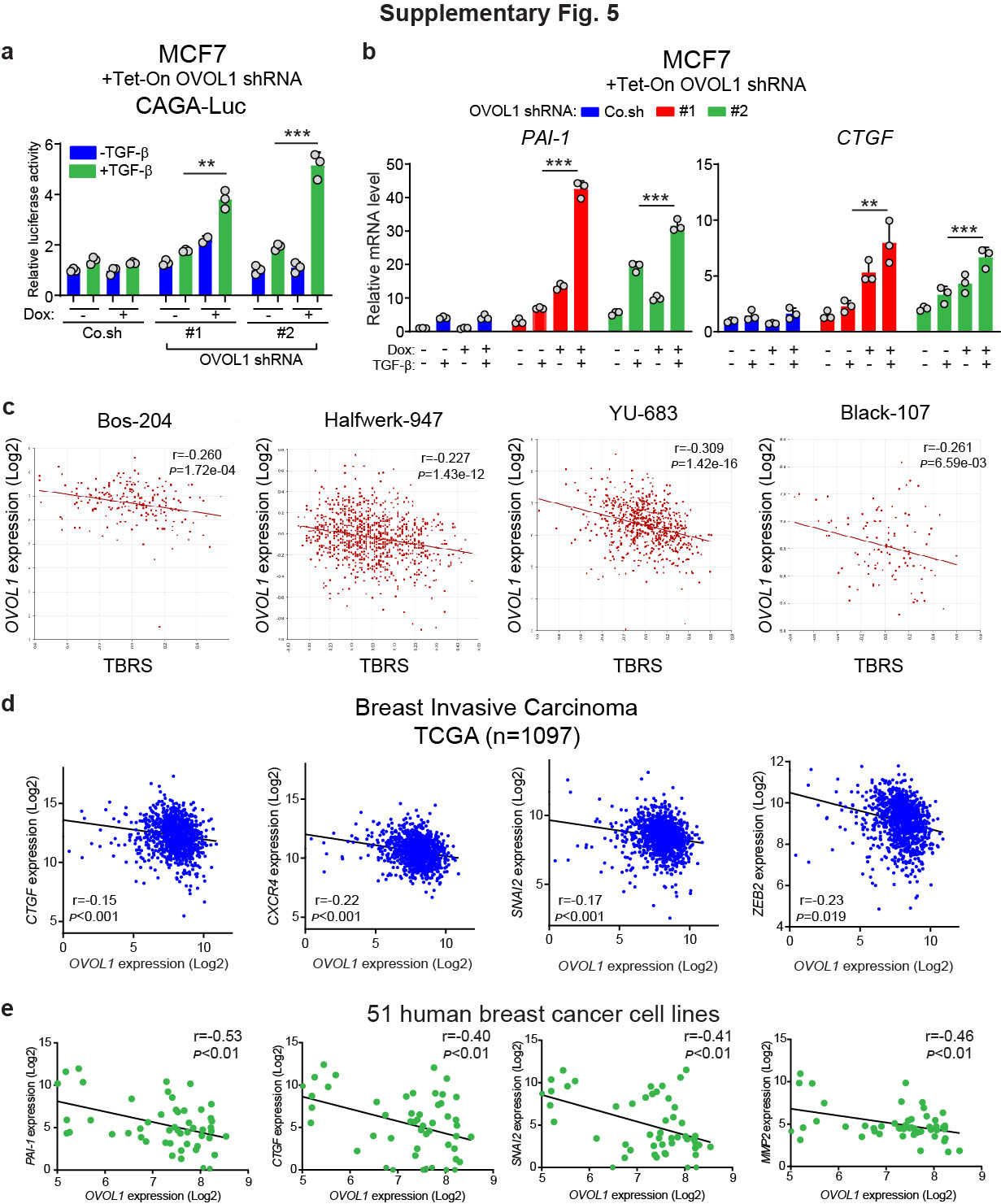
 **Supplementary Fig. 5 OVOL1 alleviates** **TGF-β pathway transduction.**

(**a**) Reporter assay for quantifying the luciferase activity in MCF7 cells stably expressing TGF-β-induced SMAD3/4-dependent CAGA-luc transcriptional reporter. Cells without (Co.sh) or with inducible OVOL1 knockdown (shRNA #1 and #2) were kept in the presence or absence of Doxycycline (Dox) for 2 days. Subsequently, cells were serum starved for 8 h and stimulated with vehicle control (-) or TGF-β (5 ng/ml) overnight. The results are expressed as mean ± SD. ** 0.001 < *p* < 0.001, *** 0.0001 < *p* < 0.001. (**b**) RT-qPCR measurement of *PAI-1* and *CTGF* expression in MCF7 cells with empty vector control (Co.sh) or inducible OVOL1 knockdown (shRNA #1 and #2). Cells were treated without or with Doxycycline (Dox) for 2 days before serum starvation overnight and stimulated with vehicle control (-) or TGF-β (5 ng/ml) for 4 h. The results are expressed as mean ± SD. ** 0.001 < *p* < 0.001, *** 0.0001 < *p* < 0.001. (**c**) Scatter plot illustrating the inverse correlation between *OVOL1* and the TGF-β response signature (TBRS) in four breast cancer datasets. Titles on top of each panels indicated the datasets in which the RNA-seq were analyzed. (**d**) Scatter plot of inverse correlation between *OVOL1* and the TGF-β/SMAD target genes (*CTGF*, *CXCR4*, *SNAI2* or *ZEB2*) in a dataset consisting of TCGA breast invasive carcinoma^1^. (**e**) Scatter plot showing correlation between *OVOL1* and the TGF-β/SMAD target genes (*PAI-1*, *CTGF*, *SNAI2* or *MMP2*) in a dataset consisting of 51 human breast cancer cell lines.


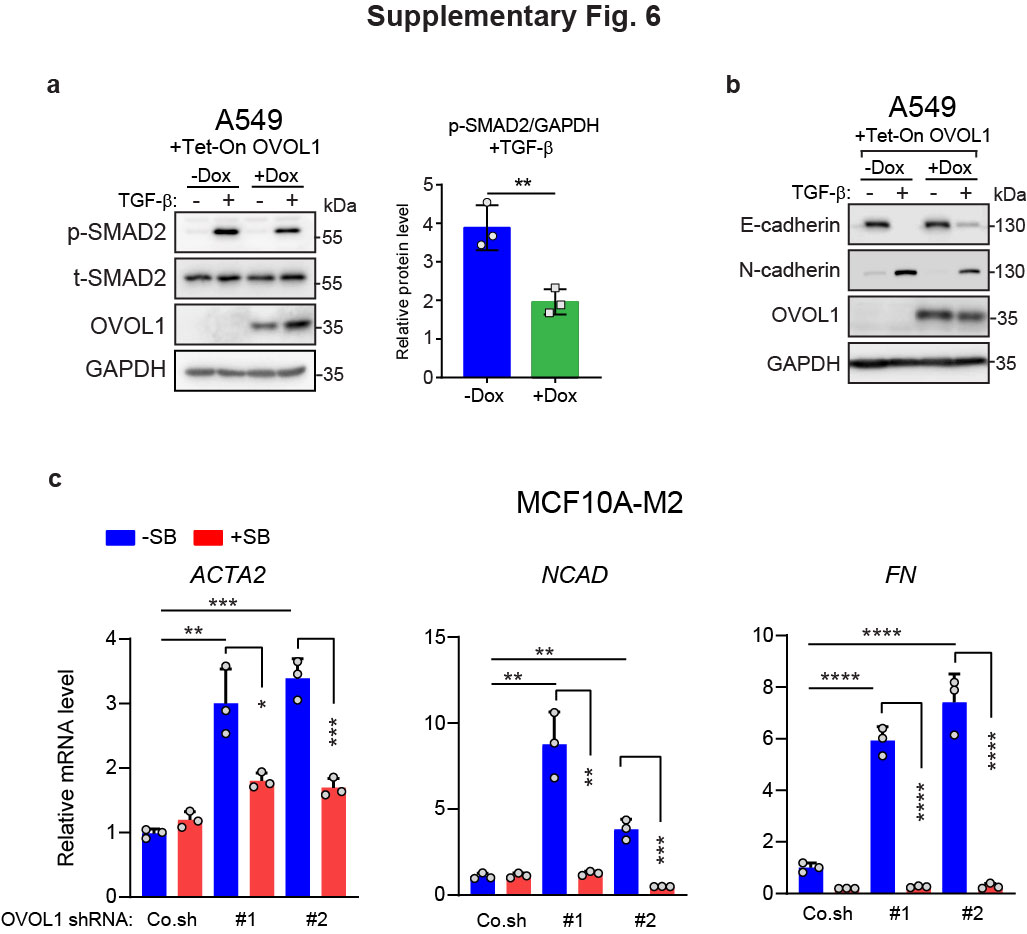
 **Supplementary Fig. 6 OVOL1 attenuates TGF-β signaling transduction.**

(**a**) Western blotting detection of the phosphorylation of SMAD2 (p-SMAD2) and total SMAD2 (t-SMAD2) in A549 cells without or with OVOL1 ectopic expression induced by Doxycycline (Dox). To control for equal loading GAPDH levels were analyzed (left panel). Cells were kept in the presence or absence of Dox for 2 days before serum starvation overnight and stimulation with vehicle control (-) or TGF-β (1 ng/ml) for 2 h. Relative protein level of p-SMAD2 was quantified from three independent sets of experiments (right panel). The results are expressed as mean ± SD. ** 0.001 < *p* < 0.001. (**b**) The expression of E-cadherin and N-cadherin detected by Western blotting in A549 cells without or with ectopic expression of OVOL1. Cells were either not treated or treated with Doxycycline (Dox) for 2 days before stimulation with vehicle control (-) or TGF-β (1 ng/ml) for 1 day. To control for equal loading GAPDH levels were analyzed. (**c**) RT-qPCR quantification of mesenchymal markers expression in MCF10A-M2 cells upon OVOL1 knockdown. Cells were kept in the presence or absence of SB431542 (SB) for 2 days. The results are expressed as mean ± SD. * 0.01 < *p* < 0.05, ** 0.001 < *p* < 0.001, *** 0.0001 < *p* < 0.001, **** *p* < 0.0001.


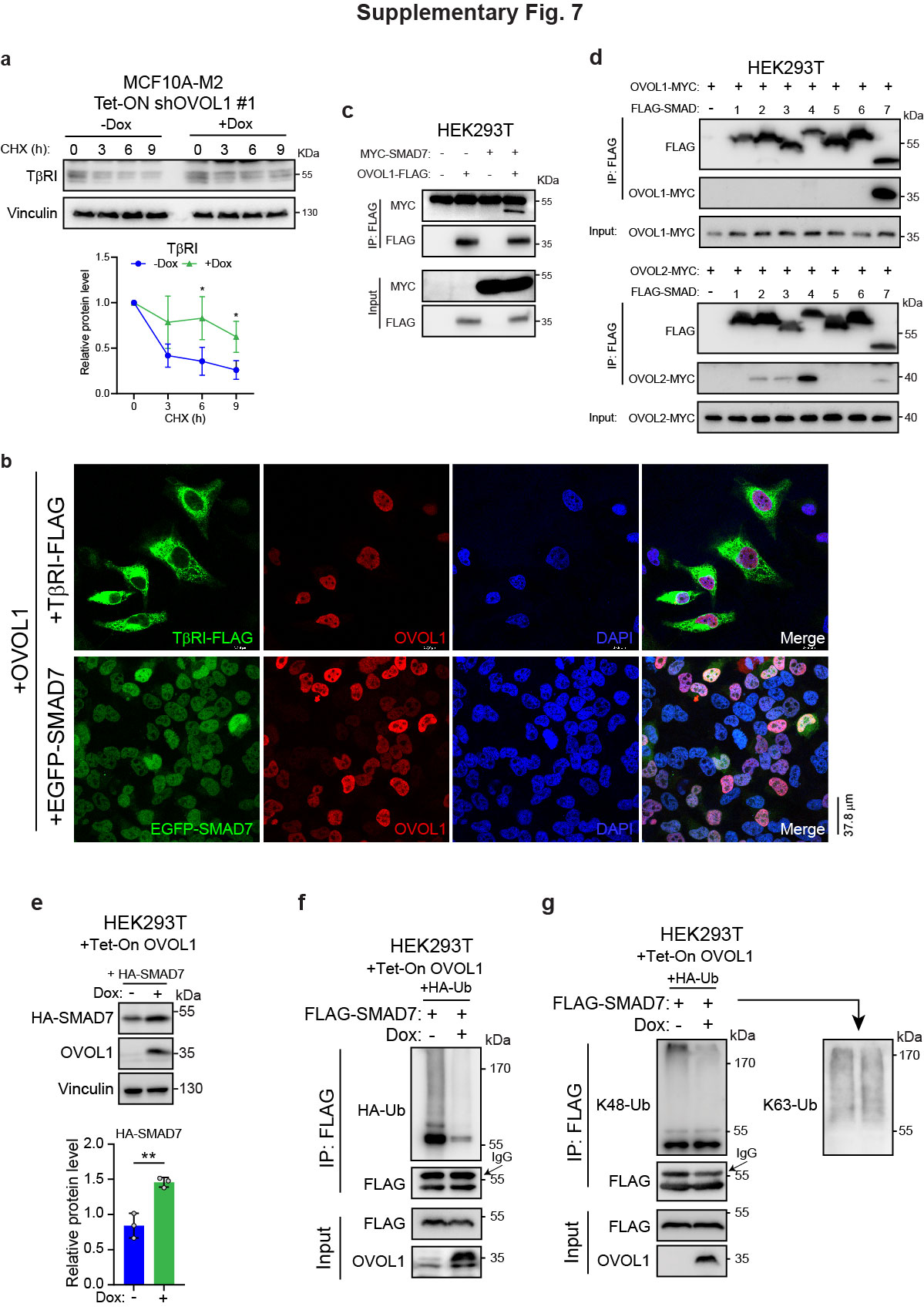
 **Supplementary Fig. 7 OVOL1 interacts with SMAD7.**

(**a**) Western blot analysis of TβRI expression levels in MCF10A-M2 cells upon OVOL1 knockdown induced by Doxycycline (Dox). Cells were either not treated or treated with Doxycycline (Dox) for 2 days followed by the stimulation of cycloheximide (CHX; 50 ug/ml) for indicated time points. Quantification of the relative TβRI protein level is shown in the lower panel. Statistical analyses were performed at indicated time points. The results are expressed as mean ± SD. * 0.01 < *p* < 0.05. To control for equal loading Vinculin levels were analyzed. (**b**) Immunofluorescence detection of TβRI-FLAG, OVOL1, EGFP-SMAD7 and 4, 6-diamidino-2-phenylindole (DAPI) staining of HeLa cells upon the transfection of indicated constructs. Scale bar, 37.8 μM. (**c**) Western blot analysis of MYC-SMAD7 and OVOL1-FLAG in whole cell lysates (Input) and immunoprecipitants derived from HEK293T cells transfected with MYC-SMAD7 and/or OVOL1-FLAG. (**d**) Western blotting detection of whole cell lysates (Input) and immunoprecipitants derived from HEK293T cells transfected with FLAG-SMADs and OVOL1-MYC or OVOL2-MYC. (**e**) HA-SMAD7 expression quantified by Western blotting in HEK293T cells with inducible OVOL1 ectopic expression (left panel). Cells were kept in the presence or absence of Doxycycline (Dox) for 1 day, followed by the transfection of HA-SMAD7. Quantification of the relative protein level of HA-SMAD7 is shown in the right panel. To control for equal loading Vinculin levels were analyzed. The results are expressed as mean ± SD. ** 0.001 < *p* < 0.01. (**f**, **g**) Western blotting analysis of whole cell lysates (Input) and immunoprecipitants derived from HEK293T cells without or with ectopic expression of OVOL1. Cells were treated without or with Doxycycline (Dox) for 1 day and then transfected with HA-Ub and FLAG-SMAD7. Total ubiquitination (**f**), K48-ubiquitination (**g**; left panel) or K63-ubiquitination (**g**; right panel) of SMAD7 was probed by indicated antibodies.


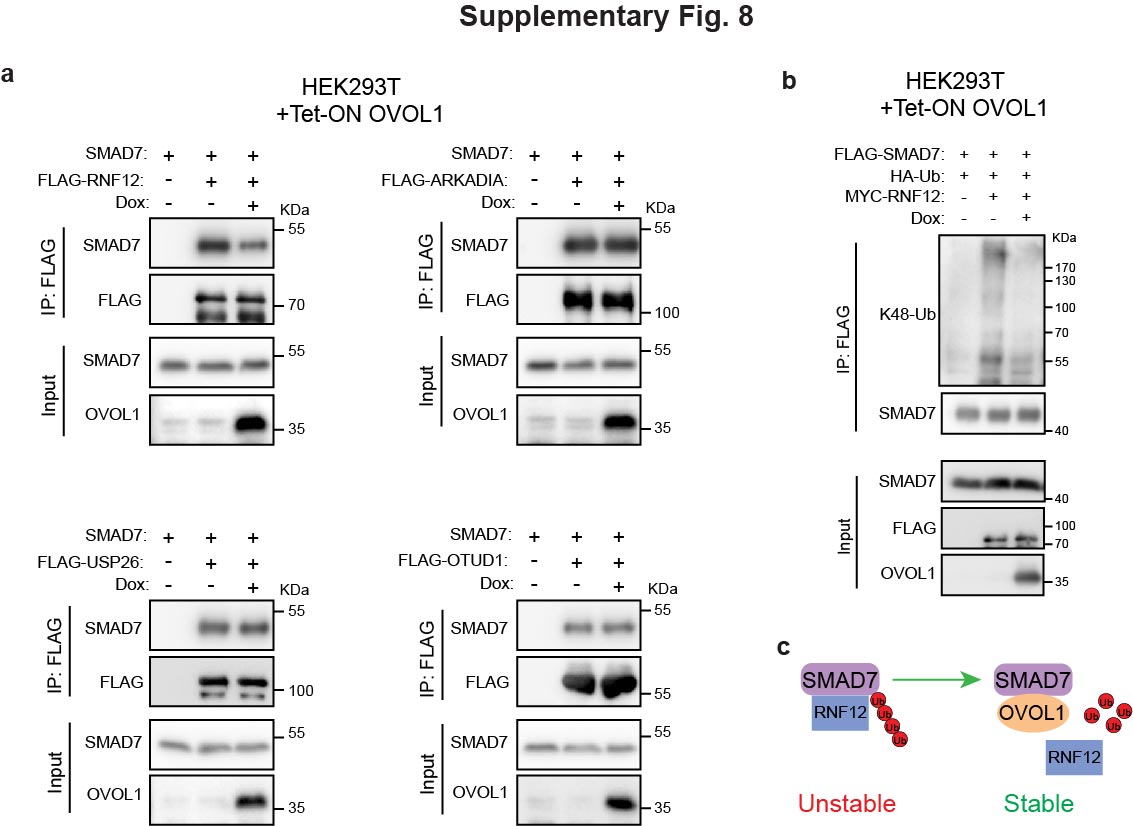
 **Supplementary Fig. 8 The effects of RNF12 on SMAD7 are diminished by OVOL1.**

(**a**) Western blot analysis of indicated proteins in whole cell lysates (Input) and immunoprecipitants derived from HEK293T cells transfected with HA-SMAD7 and FLAG-RNF12, FLAG-ARKADIA, FLAG-USP26 or FLAG-OTUD1. (**b**) Western blotting analysis of whole cell lysates (Input) and immunoprecipitants derived from HEK293T cells without or with ectopic expression of OVOL1. Cells were treated without or with Doxycycline (Dox) for 1 day and then transfected with HA-Ub, MYC-RNF12 and FLAG-SMAD7. K48-ubiquitination of SMAD7 was probed. (**c**) Schematic model of the OVOL1-induced interference of the interaction between RNF12 and SMAD7.


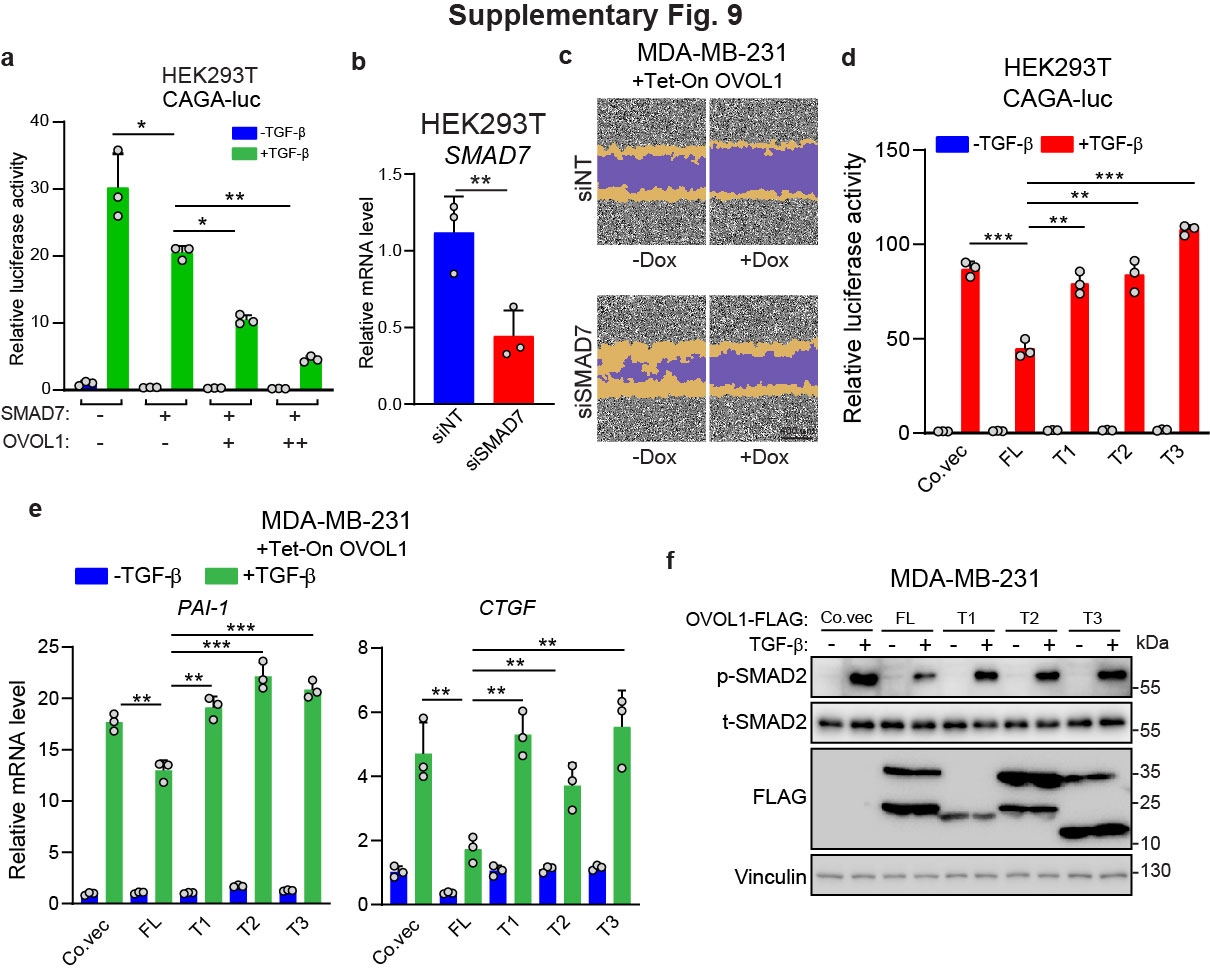
 **Supplementary Fig. 9 The inhibition of OVOL1 on TGF-β pathway is dependent on the interaction with SMAD7.**

(**a**) Reporter assay for detecting the luciferase activity in HEK293T cells transfected with TGF-β-induced SMAD3/4-dependent CAGA-luc transcriptional reporter and SMAD7 or OVOL1. Cells were stimulated with vehicle control (-) or TGF-β (5 μM). The results are expressed as mean ± SD. * 0.01 < *p* < 0.05, ** 0.001 < *p* < 0.01. (**b**) RT-qPCR measurement of *SMAD7* expression in HEK293T cells transfected with non-targeting siRNA (siNT) or siRNA targeting SMAD7 (siSMAD7). The results are expressed as mean ± SD. ** 0.001 < *p* < 0.01. (**c**) Real-time scratch assay of MDA-MB-231 cells with inducible OVOL1 expression by Doxycycline (Dox). Representative scratch wounds are shown at the end time point of the experiment. The regions of original scratches and the areas of migrating cells are colored in purple and yellow respectively. (**d**) Luciferase activity in HEK293T cells transfected with TGF-β-induced SMAD3/4-dependent CAGA-luc transcriptional luciferase reporter and empty vector control (Co.vec), FLAG tagged full-length OVOL1 (FL) or indicated OVOL1 truncation mutants (T1-T3). Cells were stimulated with vehicle control (-) or TGF-b (5 μM). The results are expressed as mean ± SD. ** 0.001 < *p* < 0.01, *** 0.0001 < *p* < 0.001. (**e**) RT-qPCR measurement of *PAI-1* and *CTGF* expression in MDA-MB-231. Cells transduced with empty vector control (Co.vec), FLAG tagged full-length OVOL1 (FL) or indicated OVOL1 truncation mutants (T1-T3) were serum starved overnight and stimulated without or with TGF-β (1 ng/ml) for 4 h. The results are expressed as mean ± SD. ** 0.001 < *p* < 0.01, *** 0.0001 < *p* < 0.001. (**f**) Western blotting analysis of the phosphorylation of SMAD2 (p-SMAD2) and total SMAD2 (t-SMAD2) in MDA-MB-231 cells. Cells transduced with empty vector (Co.vec), FLAG tagged full-length OVOL1 (FL) or indicated OVOL1 truncation mutants (T1-T3) were serum starved overnight and stimulated with vehicle control (-) or TGF-β (1 ng/ml) for 2 h. Vinculin levels were analyzed to control for equal loading.


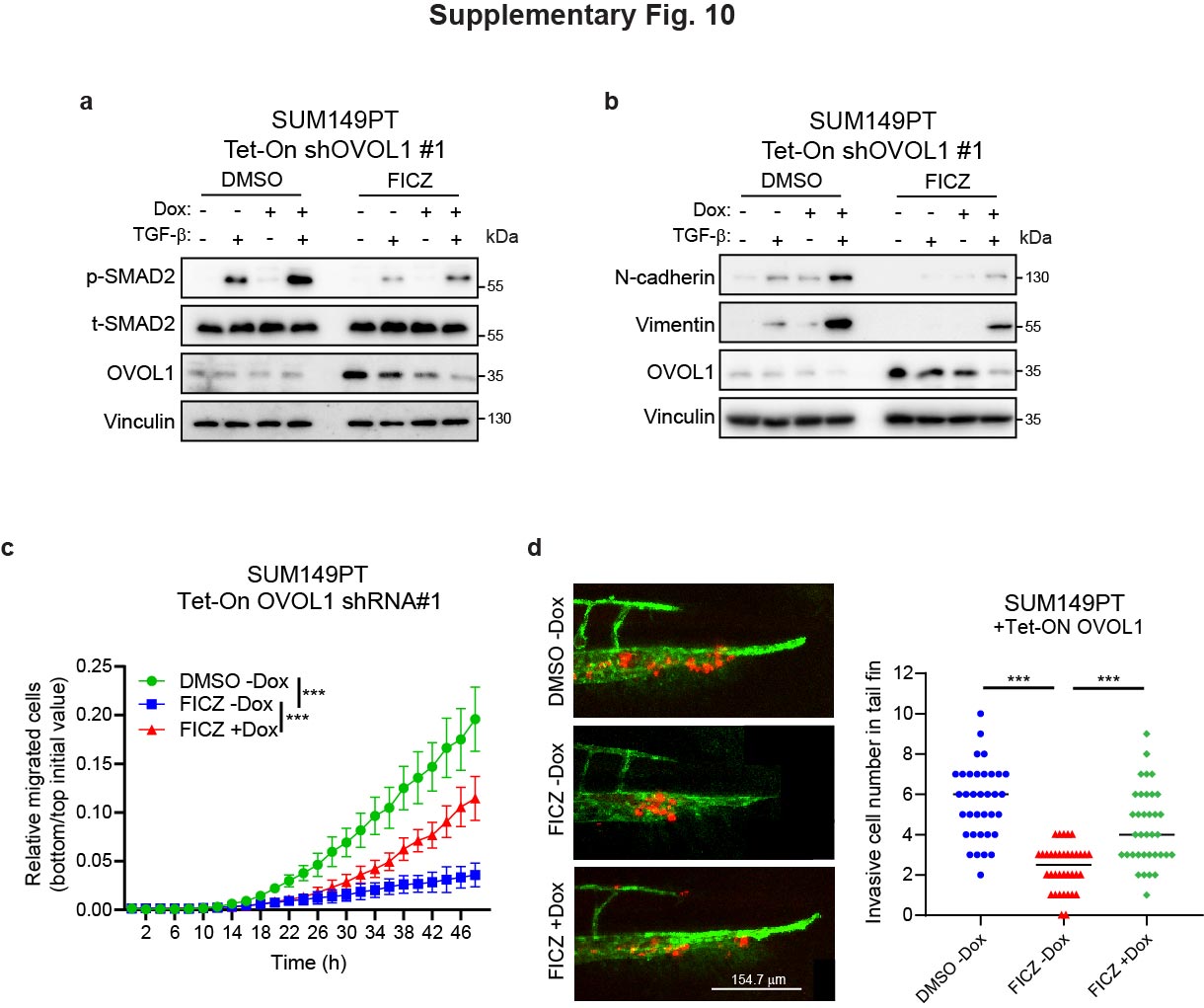
 **Supplementary Fig. 10 OVOL1 inhibits TGF-β/SMAD2 signaling and EMT, cell migration and extravasation of SUM149PT cells.**

(**a**) Western blotting measurement of the phosphorylation of SMAD2 (p-SMAD2) and OVOL1 expression in SUM149PT cells with inducible OVOL1 knockdown by shRNA #1. Cells were stimulated without or with Doxycycline (Dox) for 2 days, followed by FICZ (5 μM) treatment in serum starvation overnight before adding vehicle control or TGF-β (1 ng/ml) for another 2 h. The vehicle control DMSO was included for FICZ. Vinculin levels were analyzed to control for equal loading. (**b**) Western blotting analysis of mesenchymal markers expression in SUM149PT cells with inducible OVOL1 knockdown by shRNA #1. Cells were stimulated without or with Doxycycline (Dox) for 2 days, followed by FICZ (5 μM) treatment overnight before adding TGF-β (1 ng/ml) for 2 days. Vehicle control DMSO was included for FICZ. (**c**) IncuCyte real-time chemotaxis assay for evaluating the migration of SUM149PT cells with inducible OVOL1 knockdown by shRNA #1. Cells were pre-treated without or with Doxycycline (Dox) for 2 days before seeding into the inserts, followed by FICZ treatment. Vehicle control DMSO was included for FICZ. The results are expressed as mean ± SD. *** 0.0001 < *p* < 0.001. (**d**) *In vivo* zebrafish extravasation assay of SUM149PT cells without or with knockdown by shRNA #1. MQ water, Dox (to enable induction of the expression of OVOL1 shRNA) or FICZ (1 μM) was added to the egg water from the first day post-injection. Representative images with zoom-in of the tail fin area are shown in the left panel. Analysis of the extravasated cell numbers in indicated groups is shown in the right panel. The results are expressed as mean ± SD. *** 0.0001 < *p* < 0.001.

**Supplementary Table 1 RT-qPCR primers used in this study.**

| Gene | Sequence (5' to 3') | |
| --- | --- | --- |
| *OVOL1* | Forward | ACAGACCCCCAGAGCAGAG |
|  | Reverse | GACTGTCCCCAAGGGTCAC |
| *ID1* | Forward | CTGCTCTACGACATGAACGG |
|  | Reverse | GAAGGTCCCTGATGTAGTCGAT |
| *ID3* | Forward | CACCTCCAGAACGCAGGTGCTG |
|  | Reverse | AGGGCGAAGTTGGGGCCCAT |
| *SMAD6* | Forward | ACAAGCCACTGGATCTGTCC |
|  | Reverse | ACATGCTGGCGTCTGAGAA |
| *PAI-1* | Forward | CACAAATCAGACGGCAGCACT |
|  | Reverse | CATCGGGCGTGGTGAACTC |
| *CTGF* | Forward | TTGCGAAGCTGACCTGGAAGAGAA |
|  | Reverse | AGCTCGGTATGTCTTCATGCTGGT |
| *SMAD7* | Forward | TCCAGATGCTGTGCCCTTCC |
|  | Reverse | GTCCGAATTGAGCTGTCCG |
| *SMAD4* | Forward | CCACCAAAACGGCCATCTT |
|  | Reverse | TGGAAATGGGAGGCTGGAA |
| *TGFBRI* | Forward | ACGGCGTTACAGTGTTTCTG |
|  | Reverse | GCACATACAAACGGCCTATCT |
| *ECAD* | Forward | CCCGGTATCTTCCCCGC |
|  | Reverse | CAGCCGCTTTCAGATTTTCAT |
| *EPCAM* | Forward | CTTTATGATCCTGACTGCGATGAG |
|  | Reverse | TCAGTGTCCTTGTCTGTTCTTCTGA |
| *KRT18* | Forward | TGGCGAGGACTTTAATCTTGGT |
|  | Reverse | ACCACTTTGCCATCCACTATCC |
| *FN* | Forward | CGTCATAGTGGAGGCACTGA |
|  | Reverse | CAGACATTCGTTCCCACTCA |
| *NCAD* | Forward | CAGACCGACCCAAACAGCAAC |
|  | Reverse | GCAGCAACAGTAAGGACAAACATC |
| *VIM* | Forward | CCAAACTTTTCCTCCCTGAACC |
|  | Reverse | CGTGATGCTGAGAAGTTTCGTTGA |
| *ACTA2* | Forward | CCGGGACTAAGACGGGAATC |
|  | Reverse | TTGTCACACACCAAGGCAGT |
| *SNAIL* | Forward | ACCACTATGCCGCGCTCTT |
|  | Reverse | GGTCGTAGGGCTGCTGGAA |
| *ZEB1* | Forward | AGCAGTGAAAGAGAAGGGAATGC |
|  | Reverse | GGTCCTCTTCAGGTGCCTCAG |
| *GAPDH* | Forward | TGCACCACCAACTGCTTAGC |
|  | Reverse | GGCATGGACTGTGGTCATGAG |

**Supplementary Table 2 Antibodies used in this study.**

| Antibody | Company | Catalog number | Dilution |
| --- | --- | --- | --- |
| OVOL1 | Proteintech | 14082-1-AP | 1:1000 |
| α/β-Tubulin | Cell Signaling Technology | 2148 | 1:5000 |
| phospho-SMAD1 | Cell Signaling Technology | 9511 | 1:1000 |
| Total SMAD1 | Cell Signaling Technology | 6944 | 1:1000 |
| phospho-SMAD2 | Cell Signaling Technology | 3108 | 1:1000 |
| total SMAD2 | Epitomics | 1736-1 | 1:1000 |
| SMAD4 | Santa Cruz | sc-7966 | 1:1000 |
| E-cadherin | BD | 610181 | 1:1000 |
| N-cadherin | BD | 610920 | 1:1000 |
| Vimentin | Cell Signaling Technology | 5741 | 1:5000 |
| Fibronectin | Sigma-Aldrich | F7387 | 1:500 |
| SNAIL | Cell Signaling Technology | 3879 | 1:1000 |
| SLUG | Cell Signaling Technology | 9585 | 1:1000 |
| TβRI | Santa Cruz | sc398 | 1:1000 |
| FLAG | Sigma-Aldrich | F3165 | 1:1000 |
| HA | Santa Cruz | sc805 | 1:1000 |
| MYC | Santa Cruz | sc40 | 1:1000 |
| K48 linkage specific polyubiquitin | Cell Signaling Technology | 4289 | 1:1000 |
| K63 linkage specific polyubiquitin | Cell Signaling Technology | 5621s | 1:1000 |
| Vinculin | Sigma-Aldrich | V9131 | 1:5000 |
| GAPDH | Merck Millipore | MAB374 | 1:5000 |
| SMAD7 | R&D | MAB2029 | 1:1000 |
